# A coordinated DNA damage response promotes adult quiescent neural stem cell activation

**DOI:** 10.1371/journal.pbio.2001264

**Published:** 2017-05-10

**Authors:** Lara Barazzuol, Limei Ju, Penny A. Jeggo

**Affiliations:** 1 Genome Damage and Stability Centre, Life Sciences, University of Sussex, Brighton, United Kingdom; 2 Department of Radiation Oncology, University of Groningen, University Medical Center Groningen, Groningen, the Netherlands; 3 Department of Cell Biology, University of Groningen, University Medical Center Groningen, Groningen, the Netherlands; Osaka University, Japan

## Abstract

Stem and differentiated cells frequently differ in their response to DNA damage, which can determine tissue sensitivity. By exploiting insight into the spatial arrangement of subdomains within the adult neural subventricular zone (SVZ) in vivo, we show distinct responses to ionising radiation (IR) between neural stem and progenitor cells. Further, we reveal different DNA damage responses between neonatal and adult neural stem cells (NSCs). Neural progenitors (transit amplifying cells and neuroblasts) but not NSCs (quiescent and activated) undergo apoptosis after 2 Gy IR. This response is cell type- rather than proliferation-dependent and does not appear to be driven by distinctions in DNA damage induction or repair capacity. Moreover, exposure to 2 Gy IR promotes proliferation arrest and differentiation in the adult SVZ. These 3 responses are ataxia telangiectasia mutated (ATM)-dependent and promote quiescent NSC (qNSC) activation, which does not occur in the subdomains that lack progenitors. Neuroblasts arising post-IR derive from activated qNSCs rather than irradiated progenitors, minimising damage compounded by replication or mitosis. We propose that rather than conferring sensitive cell death, apoptosis is a form of rapid cell death that serves to remove damaged progenitors and promote qNSC activation. Significantly, analysis of the neonatal (P5) SVZ reveals that although progenitors remain sensitive to apoptosis, they fail to efficiently arrest proliferation. Consequently, their repopulation occurs rapidly from irradiated progenitors rather than via qNSC activation.

## Author summary

The response of stem cells to DNA damage is poorly understood, although there is increasing evidence that they respond distinctly to differentiated cells. We have monitored the different responses of adult neural stem and progenitor cells to exposure to X-ray irradiation. We see that progenitor cells activate apoptosis, undergo rapid proliferation arrest, and premature differentiation. However, quiescent stem cells do not activate radiation-induced apoptosis. Instead the responses of the progenitor cells promote the activation of these quiescent neural stem cells, thereby replenishing the neuroblasts. These responses and quiescent stem cell activation are dependent on the ataxia telangiectasia mutated (ATM) kinase. We propose that this coordinated response functions to remove damaged progenitor cells and to aid repopulation.

## Introduction

The response of stem cells to DNA damage plays a major role in determining the tissue response. The nature of the stem cell response following exposure to ionising radiation (IR) can underlie tissue sensitivity or resistance and may also determine sensitivity to radiation-induced carcinogenesis. Since DNA damage is encountered during normal growth and development, the response of stem cells and their immediate progenitors to exogenous damage can also provide insight into the regulation of tissue homeostasis. Here, we focus on the DNA damage response (DDR) to IR, which is of significance in evaluating the impact of radiotherapy and can influence the ageing characteristics of tissues and risk of accumulating mutations [1]. Remarkably, our knowledge of the response of stem cells to IR is fragmentary, although increasing evidence indicates that stem cells display distinct responses to somatic cells [2]. Further, recent studies have exposed, at least in certain tissues, the distinct responses of quiescent stem cells versus replicating progenitor cells [2].

The haematopoietic system is one of the best studied stem cell models, in part because haematopoietic stem cells (HSCs) and their immediate progenitors can be efficiently distinguished by fluorescence-activated cell sorting (FACS) analysis using cell-surface–specific markers. Interestingly, quiescent HSCs (qHSCs) are endowed with protective mechanisms to restrict DNA damage (e.g., a low metabolic rate and expression of ATP-binding cassette [ABC] transporter activities) [3]. Nevertheless, after IR, they inevitably incur DNA damage. Strikingly, qHSCs are resistant to apoptosis after IR, in contrast to their multipotent progenitors (MPPs) and common myeloid progenitors (CMPs), and have been reported to reenter the cell cycle to replenish the depleted progenitors [4–6]. The resistance of qHSCs to apoptosis and cell cycle reentry is p21-dependent but p53-independent, whilst apoptosis of MPPs and CMPs is p53-dependent and p21-independent [7]. Importantly, the repeated activation of HSCs out of dormancy has been reported to cause HSC attrition during ageing [6]. Quiescent mammary stem cells (MaSCs), crypt stem cells, and bulge stem cells (BSCs) of the hair follicle similarly display resistance to apoptosis in contrast to their respective transit amplifying progenitors (TAPs), which sensitively activate apoptosis [7,8]. Quiescent cells rely on DNA nonhomologous end-joining (NHEJ), which is perceived to be error-prone [5]. However, hair follicle BSCs have been proposed to up-regulate NHEJ as a mechanism to enhance recovery to IR [8]. Additionally, to help maintain genomic stability, it has been proposed that HSCs can differentiate if they harbour excess DNA damage [9,10].

**Fig 1:**
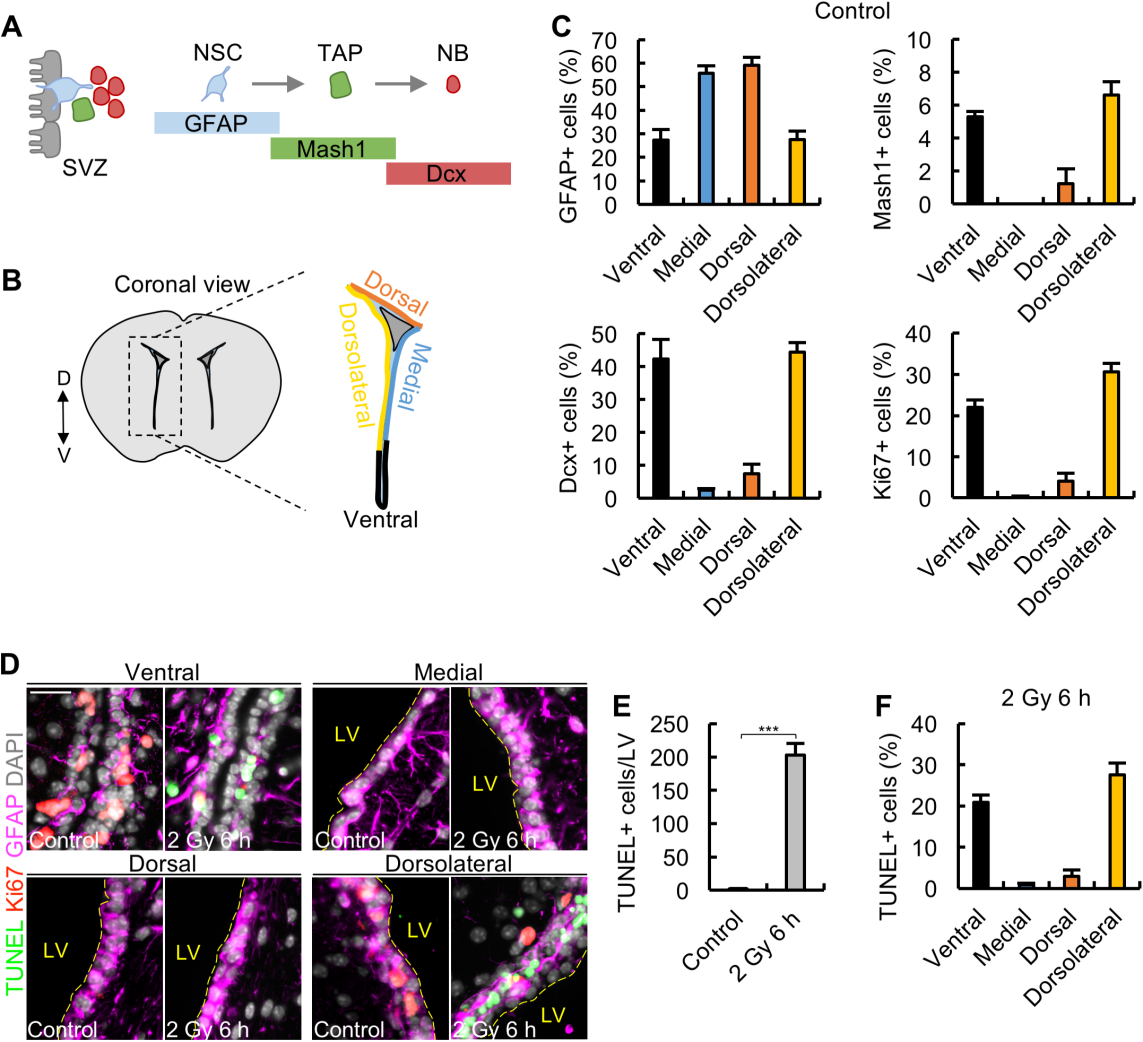
Apoptosis after 2 Gy ionising radiation (IR) only arises in the 2 subdomains containing progenitor cells. (A) Scheme showing the lineage of neural stem cells (NSCs) within the subventricular zone (SVZ). Glial Fibrillary Acidic Protein (GFAP)^+^ neural stem cells (NSCs; light blue) are predominantly quiescent. 5%–10% are activated, however, and can produce mammalian Achaete-scute homologue 1 (Mash1)^+^ transit amplifying progenitors (TAPs; green), which in turn give rise to doublecortin^+^ (Dcx^+^) neuroblasts (NBs; red). 50% of NBs are proliferative. NSCs, TAPs, and NBs are also called type B, C, and A cells, respectively. (B) Graphical illustration showing the subdomain structure around the lateral ventricle. The lineage of cells arising from these subdomains is shown in S1A Fig. The colour code (black, ventral; blue, medial; orange, dorsal; yellow, dorsolateral) will be used throughout. (C) Quantification of GFAP^+^, Mash1^+^, Dcx^+^, and Ki67^+^ cells within the 4 subdomains of untreated control mice. In this and all subsequent figures, the percentages are normalised to the number of 4′,6-diamidino-2-phenylindole (DAPI) positive cells. (D) Images showing GFAP^+^ cells (magenta), Ki67^+^ cells (red), terminal deoxynucleotidyl transferase-mediated dUTP nick end-labelling (TUNEL)^+^ cells (green), and DAPI staining (grey) within the 4 subdomains. Separate colour channels are given in S1E Fig. Apoptosis is only observed in the dorsolateral and ventral subdomains. (E) Quantification of TUNEL^+^ cells in the entire lateral ventricle (LV) in control and irradiated mice at 6 h post 2 Gy. (F) Quantification of TUNEL^+^ cells within each subdomain at 6 h post 2 Gy. Experiments were carried out on 3-month-old mice and represent the mean ± SEM of *n* ≥ 3 mice for each condition. Scale bars, 25 μm. Student *t* test, *** *P* < 0.001. Underlying data can be found in the S1 Data file.

Here, we focus on neural stem cells (NSCs) located in the subventricular zone (SVZ), the largest germinal region in the adult forebrain, which provides neurogenesis into adulthood [11]. Adult NSCs, designated type B cells, have an astrocytic phenotype and express Glial Fibrillary Acidic Protein (GFAP) [11]. They are relatively quiescent, but around 10% are activated to proliferate. They undergo symmetric division to generate further NSCs as well as asymmetric division to generate type C TAPs, which express mammalian Achaete-scute homologue 1 (Mash1). TAPs also undergo symmetric division to expand in number and asymmetric division to generate doublecortin^+^ (Dcx^+^) neuroblasts (NBs) (type A cells) (Fig 1A). NBs migrate through the rostral migratory stream to the olfactory bulb and terminally differentiate into subpopulations of mature interneurons (S1A Fig). TAPs divide 3 times, and NBs divide once or twice [12]. Several studies have examined the response of these distinct cell types to DNA damage. Exposure to the antimitotic agent, cytosine-β-d-arabinofuranoside, resulted in the elimination of proliferating (type A and C) progenitors, but type B cells remained and promoted repopulation [13]. In the rat brain, 5 Gy IR induced apoptosis in the subependymal layer, representing cells adjacent to the lateral ventricle, with subsequent repopulation indicating a radiation-resistant stem cell subset [14]. Using a FACS approach, a more recent study identified and quantified quiescent NSCs (qNSCs) and proliferating progenitors and observed loss of proliferating progenitors with subsequent exit of qNSCs from dormancy after IR exposure [15]. Collectively, these studies provide evidence that the SVZ may respond to DNA damage in a similar manner to that observed in the HSC compartment, although details of which cells die by apoptosis, the underlying mechanism, and the coordination with proliferation in vivo is lacking.

The adult SVZ has been shown to be spatially heterogeneous and can be divided along the dorso-ventral axis into subdomains designated as dorsal, medial, ventral, and dorsolateral [16–18](Fig 1B). Each subdomain has a distinct embryonic origin and gives rise to different olfactory bulb neuronal subtypes (S1A Fig). Additionally, the subdomains differ in their proliferative/quiescent status. Most studies to date examining the response to IR in the SVZ have not considered the topographical division into different subdomains. The subdomain organization, however, could be informative if they differ in the degree of proliferation. A further aspect affecting NSC proliferative status is postnatal age. Multiple NBs are generated in newborn mice, but this rapidly diminishes with progression into adulthood, which is accompanied by an increased level of qNSCs [19]. Significantly, young mice and people are sensitive to IR-induced neuronal carcinogenesis in contrast to the resistance of adults [20–22]. Whether neonatal and adult mice differ in their response to IR has not been examined yet might be significant in evaluating the specific IR-induced cancer predisposition of the juvenile brain.

In the first part of our study, we examine the response to IR within the adult SVZ, taking advantage of knowledge of the subdomain structure. Importantly, our analysis is undertaken in vivo allowing us to assess the response in each subdomain under the in vivo proliferation state. We then exploit this insight to evaluate distinctions in the response between adult and neonatal mice. This approach proved to be insightful in assessing the distinct responses of NSCs, TAPs, and NBs. We demonstrate that progenitors (TAPs and NBs) but not NSCs (quiescent and activated) undergo apoptosis and characterize 2 novel responses, namely proliferation arrest and progenitor marker loss. We show that these responses are ataxia telangiectasia mutated (ATM)-dependent and drive qNSC activation, which replenishes the pool of NBs. The ability to activate apoptosis is cell type- rather than proliferation-dependent and does not appear to be driven by distinctions in DNA damage induction or repair capacity. We propose that the benefit of apoptosis is not to confer sensitive cell death but rather to remove progenitor cells to aid repopulation, a beneficial feature since proliferating cells are likely to be the ones more sensitive to cancer-driving genetic changes. Importantly, our analysis of these responses in neonatal mice reveals distinctions to the response in adults, demonstrating that progenitor repopulation occurs more rapidly from irradiated progenitors that fail to arrest proliferation rather than via qNSC activation.

## Results

### Neural progenitors undergo apoptosis, whilst qNSCs are apoptosis resistant

Delineation of the subdomains within the lateral ventricle is shown in Fig 1B. First, we assessed the percentage of NSCs (type B cells; GFAP^+^), TAPs (type C cells; Mash1^+^), and NBs (type A cells; Dcx^+^) in the adult SVZ (defined as 3-month-old mice) in each subdomain (Fig 1C). We observed high (>90%) costaining of GFAP with Sox2 and Nestin in all 4 subdomains. Thus, to aid clarity, we have designated all GFAP^+^ cells as NSCs, although they may encompass a small number of astrocytes. Hereafter the term neural progenitors will be used to represent TAPs and NBs. Cell numbers are expressed as the percentage of 4′,6-diamidino-2-phenylindole (DAPI)^+^ cells within each subdomain. GFAP^+^ cells are located in all 4 subdomains, with higher levels in the medial and dorsal regions (Fig 1C). Consistent with the more quiescent nature of the medial and dorsal domains, there were very low (or undetectable) numbers of neural progenitors (Mash1^+^ TAPs and Dcx^+^ NBs) in these 2 subdomains; indeed, the progenitors were predominantly restricted to the ventral and dorsolateral domains (Fig 1C and 1D). To examine proliferation within these subdomains, we assessed the distribution of the proliferation marker, Ki67. Consistent with the proliferative nature of the progenitors (TAPs and NBs), Ki67^+^ cells were also predominantly localized to the ventral and dorsolateral domains, with 20%–30% of the cells in these 2 subdomains being Ki67^+^ (Fig 1C and 1D). At 6 h following exposure to 2 Gy, apoptotic cells (assessed by terminal deoxynucleotidyl transferase-mediated dUTP nick end-labelling [TUNEL] staining) were observed in the lateral ventricle (Fig 1E) with little or no apoptosis in the surrounding differentiated tissue (S1B Fig). Strikingly, the distribution of apoptotic cells within the subdomains revealed that apoptosis occurred almost uniquely in the ventral/dorsolateral regions where the proliferating progenitors were located (Fig 1F).

Collectively, these findings based on spatial analysis of the subdomains demonstrate that the GFAP^+^ cells, which are abundant in the medial/dorsal regions, are resistant to apoptosis. Additionally, the findings provide strong evidence that it is the neural progenitors that undergo apoptosis. These findings are consistent with studies on the haematopoietic system and provide initial evidence that it is specifically the neural progenitor cells (TAPs and/or NBs) that succumb to apoptosis [7].

### Sensitivity to apoptosis reflects cell type not proliferative status

**Fig 2:**
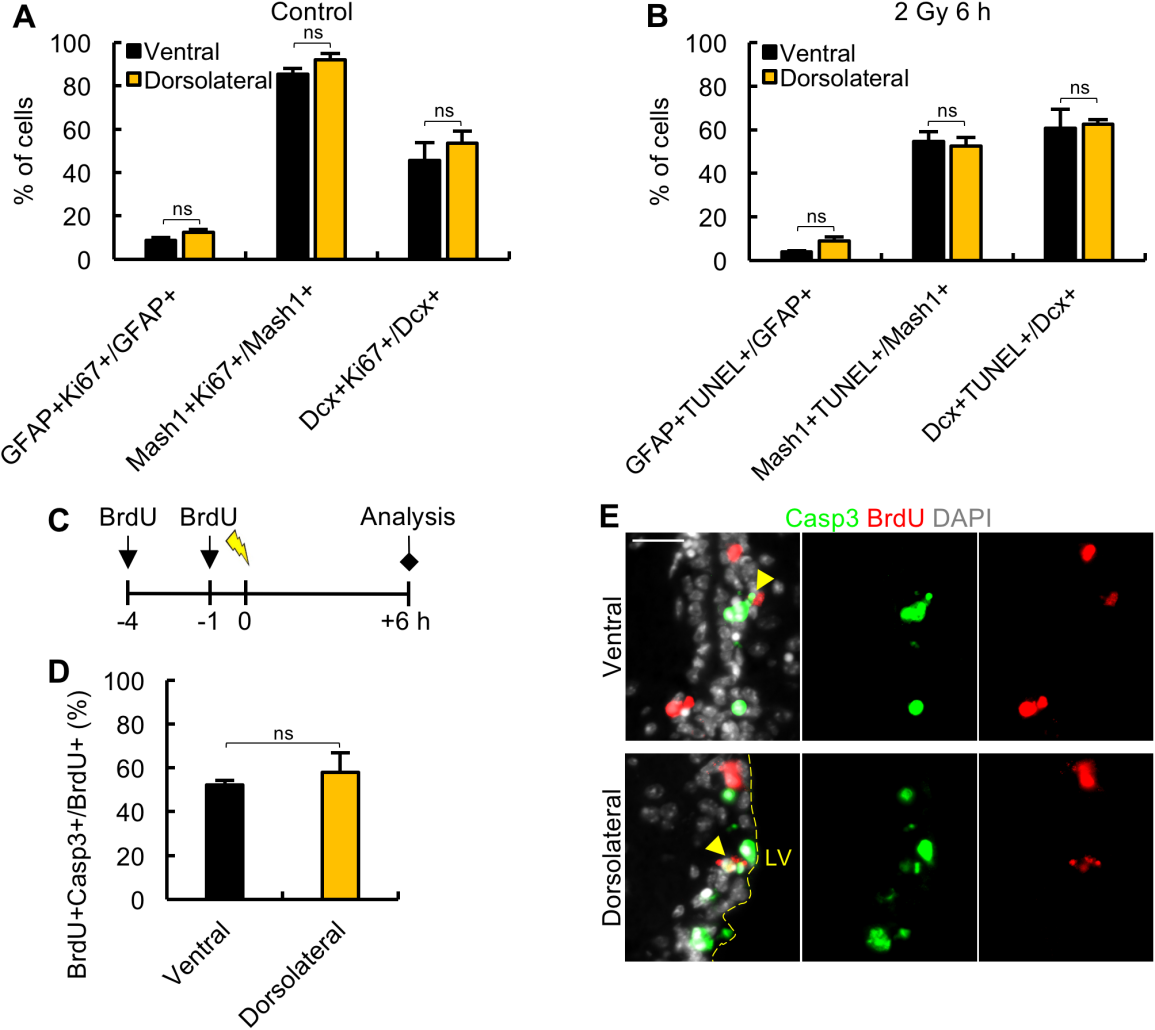
Fifty percent of the progenitor cells undergo apoptosis after 2 Gy regardless of proliferative status. (A) The percentage of Ki67^+^ cells of each cell type analysed (Glial Fibrillary Acidic Protein [GFAP]^+^, mammalian Achaete-scute homologue 1 [Mash1]^+^ and doublecortin^+^ [Dcx^+^]) in the ventral and dorsolateral subdomains of the subventricular zone (SVZ). Only low numbers of Ki67^+^ cells were detected in the medial and dorsal subdomains (Fig 1C). (B) The percentage of terminal deoxynucleotidyl transferase-mediated dUTP nick end-labelling (TUNEL)^+^ cells of each cell type analysed at 6 h post 2 Gy. (C) A scheme showing the protocol to assess percentage of replicating cells undergoing apoptosis using bromodeoxyuridine (BrdU) incorporation to assess replication. Mice were given 2 injections of BrdU at 4 h and 1 h prior to exposure to 2 Gy IR and assessed for apoptosis (cleaved caspase-3 [Casp3^+^]) and/or BrdU incorporation 6 h later. (D) The quantification of BrdU^+^Casp3^+^ cells out of the total BrdU^+^ cells. Assessment of Casp3^+^BrdU^+^ cells out of the total Casp3^+^ cells is shown in S2B Fig. (E) The representative images of labelled cells for the experiment shown in panels C-D. Black and yellow columns show analysis of the ventral and dorsolateral subdomains, respectively. Experiments were carried out on 3-month-old mice and represent the mean ± SEM of *n* ≥ 3 mice for each condition. Scale bars, 25 μm. Student *t* test, ns = not significant. Underlying data can be found in the S1 Data file.

To further evaluate the nature of cells undergoing apoptosis, we assessed the percentage of each cell type expressing Ki67 in the ventral or dorsolateral regions. Consistent with the quiescent nature of NSCs, only approximately 5%–10% of GFAP^+^ NSCs were Ki67^+^ (Fig 2A). Additionally, consistent with cell lineage analysis, approximately 80%–90% of the Mash1^+^ TAPs and 50% of Dcx^+^ NBs expressed Ki67, respectively (Fig 2A). From this quantification and that in Fig 1C, the distribution of Ki67^+^ cells in the ventral and dorsolateral regions showed that Dcx^+^ NBs represent the predominant progenitor cell type in the ventral and dorsolateral domains (S1C Fig). Assessment of the percentage of each cell type undergoing apoptosis revealed that approximately 50%–60% of Mash1^+^ or Dcx^+^ cells were TUNEL^+^ at 6 h post IR (Fig 2B). There were very few GFAP^+^TUNEL^+^ cells, supporting the notion that qNSCs are resistant to apoptosis (Fig 2B). As expected from the cell distribution, the predominant cell type undergoing apoptosis were Dcx^+^ NBs, which represented 60%–70% of the apoptotic cells at 6 h post IR (S1D Fig).

These findings demonstrate that both progenitor cell types (TAPs and NBs), which are located predominantly in the dorsolateral and ventral subdomains, undergo apoptosis after 2 Gy IR. In these domains, we observed that approximately 50% of Dcx^+^ NBs are Ki67^+^, and approximately 50% Dcx^+^ NBs undergo apoptosis after 2 Gy. Thus, after 2 Gy, either 100% of the Dcx^+^Ki67^+^ NBs (i.e., actively proliferating Dcx^+^ NBs) succumb to apoptosis, or 50% of all Dcx^+^ NBs undergo apoptosis irrespective of proliferative status. Thus, we sought to address the relationship between apoptotic sensitivity and proliferative status. We did not wish to use the Ki67 marker to assess this relationship since, as confirmed below, we predicted that this marker might be affected by IR exposure at 6 h post IR (when apoptosis is quantified). To address whether all progenitors can become apoptotic or only those that are actively proliferating, we prelabelled proliferating cells with BrdU at 4 h and 1 h prior to exposure to 2 Gy IR and at 6 h post IR assessed the level of apoptotic BrdU^+^ cells out of the total BrdU^+^ cells (Fig 2C). For technical reasons, apoptosis was assessed using cleaved Caspase-3 (Casp3) staining for this analysis, but control experiments verified that a similar level of apoptosis was detected by TUNEL or Casp3 staining (S2A Fig). Significantly, at 6 h post IR, all BrdU^+^ cells were localized to the ventral/dorsolateral domains but only 50%–60% (depending on the precise subdomain) were Casp3^+^ (Fig 2D and 2E). This demonstrates that 50% of the progenitors, despite being BrdU labelled immediately prior to IR (i.e., actively proliferating), do not undergo apoptosis. This strongly suggests that 50% of progenitors undergo apoptosis after 2 Gy regardless of their proliferative status. (Note also that we observed that approximately 30% of the Casp3^+^ cells were BrdU^+^, which was consistent with the conclusion above but is less informative because the BrdU labelling may not detect all proliferating cells [S2B Fig]). This conclusion is also consistent with the finding that, although approximately 80% of Mash1^+^ cells are Ki67^+^, only 50% are TUNEL^+^ (Fig 2A and 2B).

This analysis provides strong evidence that the sensitivity to apoptosis is a feature of the progenitor cell type (Mash1^+^ or Dcx^+^) rather than being a direct consequence of proliferation per se. Nonetheless it is the neural progenitors, the proliferative cells in the SVZ, which are apoptotic-sensitive.

### Proliferative arrest and progenitor marker loss represent additional IR-induced responses distinct from apoptosis

**Fig 3:**
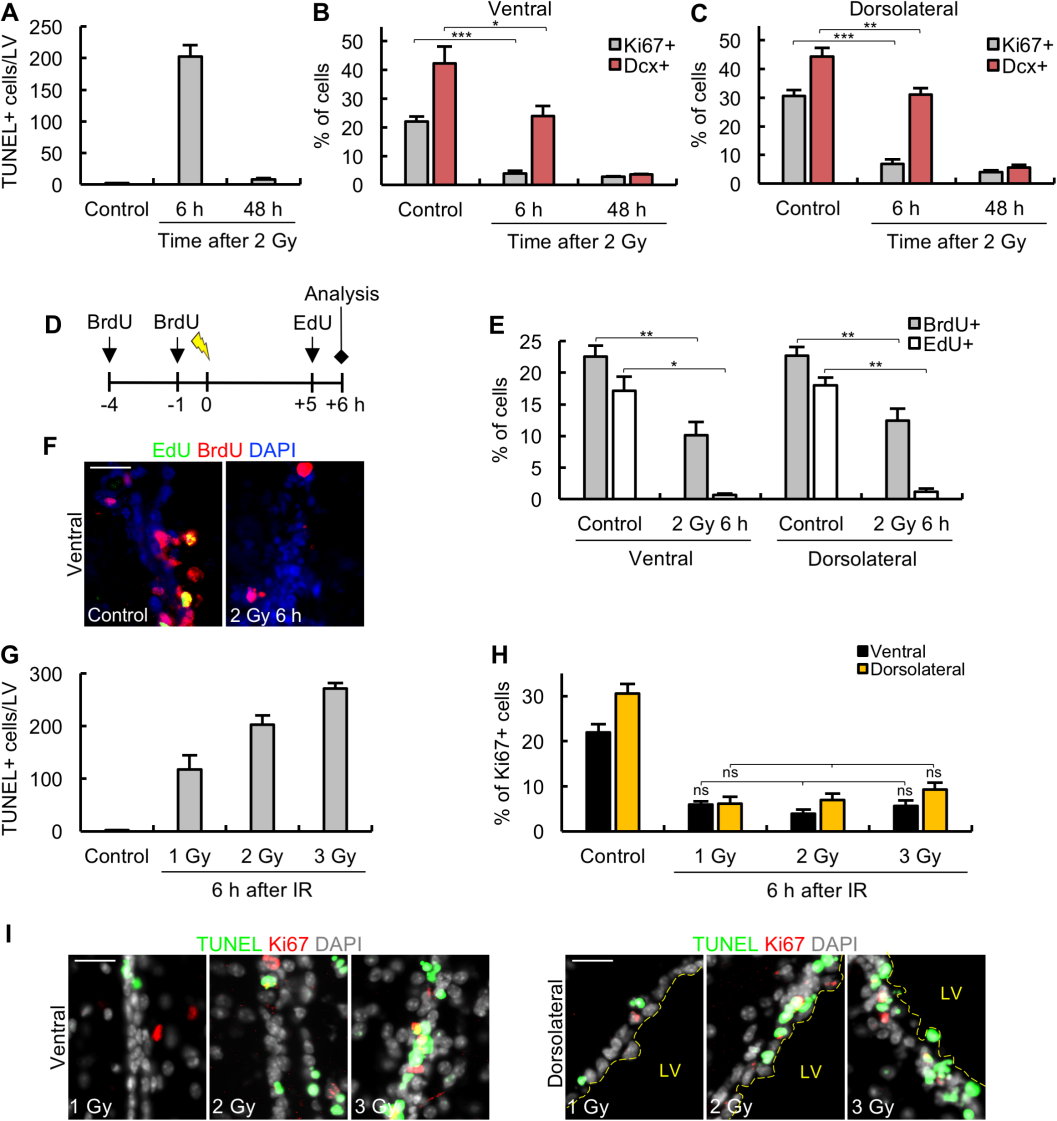
Arrest of proliferation and progenitor marker loss are additional DNA damage responses to 2 Gy ionising radiation (IR). (A) Apoptosis ceases by 48 h post 2 Gy. Apoptosis in the lateral ventricle (LV) was assessed by terminal deoxynucleotidyl transferase-mediated dUTP nick end-labelling (TUNEL) staining at 6 h and 48 h post 2 Gy and quantified as the total number of TUNEL^+^ cells per LV. (B-C) Proliferation (Ki67) and the neurobloast (NB) marker (doublecortin [Dcx]) are diminished by 48 h post 2 Gy. The quantification of Ki67^+^ and Dcx^+^ cells in the ventral (panel B) and dorsolateral (panel C) subdomains at 6 h and 48 h post 2 Gy. Grey columns, Ki67; red columns, Dcx. (D) A schematic showing the protocol for bromodeoxyuridine (BrdU)/ethynyl deoxyuridine (EdU) labelling of replicating cells post 2 Gy. (E) The assessment of percentage of BrdU^+^ or EdU^+^ cells in the ventral and dorsolateral subdomains in unirradiated control mice or at 6 h following 2 Gy. (F) The representative images from the experiment shown in panel D-E. (G-I) Dose-response analysis of apoptosis and Ki67^+^ cells, respectively. Adult mice were exposed to the indicated dose of IR and quantified for apoptosis (TUNEL) in the LV (panel G) or Ki67^+^ cells in the ventral or dorsolateral subdomains (panel H) at 6 h post IR. Panel I shows representative images. For panels G and H, black and yellow columns show analysis of the ventral and dorsolateral subdomains, respectively. Experiments were carried out on 3-month-old mice and results represent the mean ± SEM of *n* ≥ 3 mice for each condition. Scale bars, 25 μm. Student *t* test, * *P* < 0.05, ** *P* < 0.01, *** *P* < 0.001, ns = not significant. Underlying data can be found in the S1 Data file.

To gain insight into the downstream consequences of IR exposure, we examined the number of Dcx^+^ and Ki67^+^ cells at longer times post IR in the ventral and dorsolateral domains. For this analysis, we focused on Dcx^+^ cells since Mash1^+^ TAPs represent a small subset (approximately 6%) of progenitors. First, we will discuss the events taking place up to 48 h post IR. Analysis of TUNEL^+^ cells showed that apoptosis peaked at 6 h post 2 Gy X-rays with no detectable apoptosis at 48 h (Fig 3A). Strikingly, the number of Ki67^+^ cells in the ventral and dorsolateral regions was dramatically diminished at 6 h and 48 h post IR (Fig 3B and 3C). Since apoptotic cells are detectable at 6 h post IR (and 50% of the Dcx^+^ cells at 6 h are TUNEL^+^), this strongly suggests that the Ki67 marker is lost following IR exposure in a manner that cannot be attributed to cell loss by apoptosis. Somewhat distinct to these findings, we found that the percentage of Dcx^+^ cells was less dramatically decreased at 6 h but by 48 h was very low (Fig 3B and 3C). Notably, in the dorsolateral domain, although >50% of the Dcx^+^ cells retain the NB marker at 6 h post IR, very few display the Ki67 marker demonstrating the distinct kinetics between these 2 responses. Additionally, although only 50% of Dcx^+^ cells appear to undergo apoptosis, by 48 h, very few express the Dcx^+^ marker. This strongly suggests that there is a striking loss of progenitor cells expressing the Dcx marker between 6 h and 48 h post IR. Significantly, we observed only a 20%–25% decrease in overall DAPI-staining cells and a similar decrease in subdomain thickness at 48 h post IR, which is consistent with the fraction of total cells that undergo apoptosis; furthermore, these changes are only observed in the subdomains that undergo apoptosis (S2C and S2D Fig). Since NBs represent approximately 50% of the total cells in the ventral and dorsolateral regions (Fig 1C), this supports the notion that they do not all undergo apoptosis but, as a distinct response, cease proliferation and lose their defining marker.

Ki67 is an indirect cell surface marker of proliferation. We also examined proliferation using ethynyl deoxyuridine (EdU) incorporation as a more direct monitor of replicating cells. Using the approach described in Fig 3D, we labelled cells replicating prior to IR exposure using BrdU (added at 4 h and 1 h prior to IR) and monitored ongoing replication from 5 h to 6 h post IR by EdU incorporation. We assessed the fraction of BrdU^+^ or EdU^+^ cells with or without IR exposure. These findings confirmed a dramatic arrest in replication (EdU^+^ cells) between 5 h to 6 h post IR exposure (Fig 3E and 3F). We also observed an approximately 2-fold loss of BrdU^+^ cells by 6 h post IR, which may be a consequence of the onset of DNA degradation during apoptosis and/or cell division and dilution of the BrdU^+^ signal in the unirradiated control cells.

Additionally, to provide further evidence for the distinct nature of apoptosis and proliferation arrest, we carried out a dose-response analysis monitoring the level of TUNEL^+^ and Ki67^+^ cells reasoning that they may show a distinct dose-response relationship. Indeed, while we observed a linear dose response for apoptosis up to 3 Gy, we observed a loss of Ki67^+^ cells even after exposure to 1 Gy (Fig 3G–3I). Thus, we conclude that IR exposure to ≥1 Gy causes arrest of proliferation in a nonapoptotic-dependent manner.

Collectively, these findings identify 2 novel responses to IR exposure in the SVZ, namely the rapid inhibition of proliferation followed by a loss of cells bearing the Dcx marker.

### Radiation-induced DNA damage promotes differentiation of NBs

We next aimed to gain insight into the basis underlying the loss of the NB (Dcx) marker. The number of DAPI cells in the SVZ only diminished by 20%–25% at 48 h, a level entirely attributable to apoptosis. If loss of the Dcx marker represents elimination of immature neurons (e.g., by movement out of the SVZ), it must be compensated by movement of cells into the SVZ. We therefore examined whether the number of microglia, the most likely candidate cell type moving into the irradiated SVZ, increases after IR (S3 Fig). Following staining for the microglial marker, Iba1, we did not observe any significant increase in either the ventral or dorsolateral subdomains, although there was a small but nonsignificant increase when the entire lateral ventricle was analysed (S3A–S3C Fig). There was no difference in the percentage of Iba1^+^ cells in the SVZ or the differentiated, nonapoptotic isocortex (S3D and S3E Fig).

**Fig 4:**
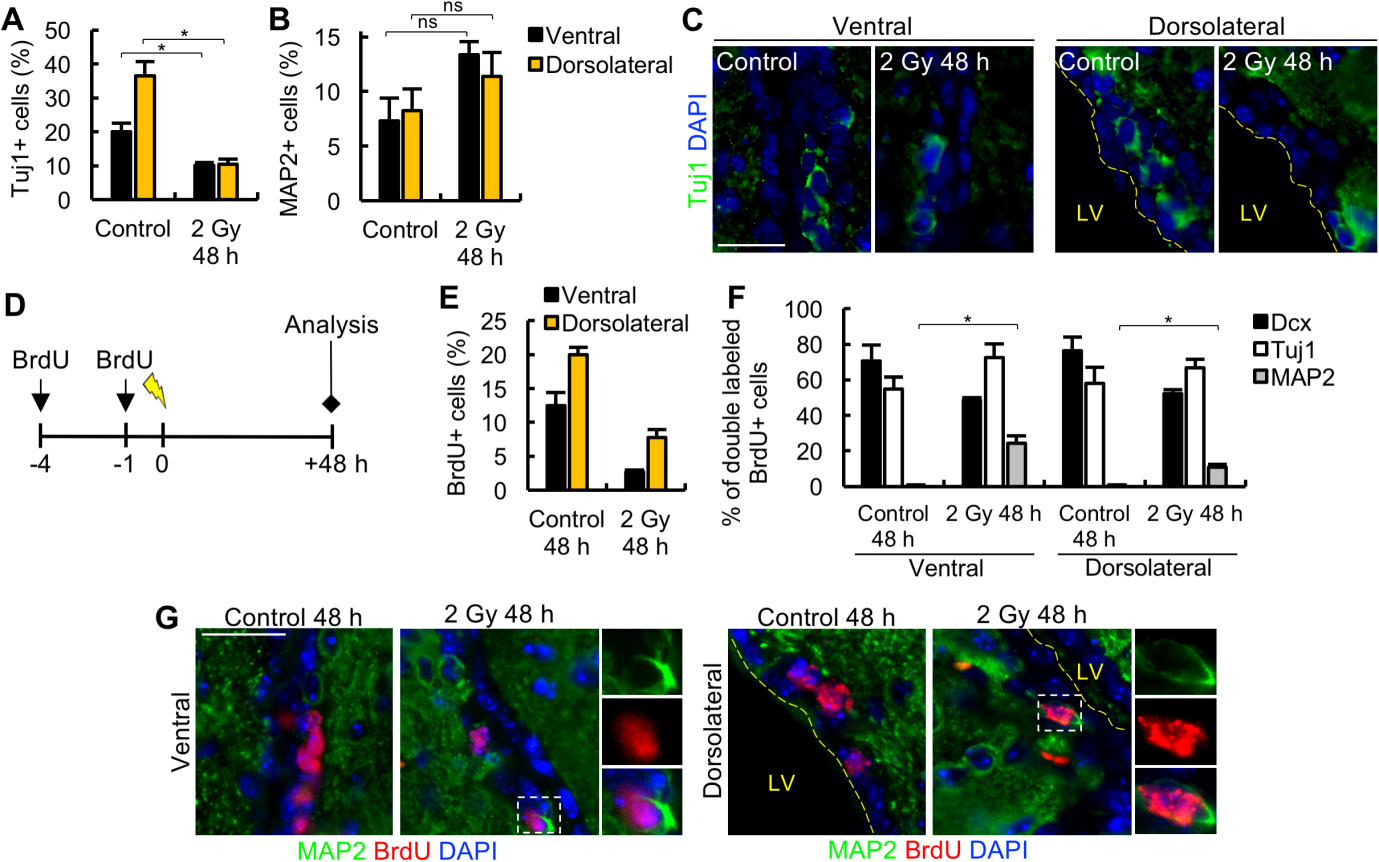
DNA damage-induced neuronal differentiation. (A-B) The quantification of the percentage of neuron-specific class III β-tubulin(Tuj1)^+^ and microtubule-associated protein 2 (MAP2)^+^ cells in the ventral and dorsolateral subdomains of control and irradiated mice at 48 h post 2 Gy. (C) The representative images for the experiment shown in panel A, showing loss of Tuj1^+^ cells at 48 h following IR. (D) A schematic showing the protocol for bromodeoxyuridine (BrdU) labelling. (E) Percentages of BrdU^+^ cells in the ventral and dorsolateral subdomains in unirradiated control mice and at 48 h following 2 Gy. (F) Percentages of Dcx^+^BrdU^+^, Tuj1^+^BrdU^+^, and MAP2^+^BrdU^+^ cells out of the total BrdU^+^ cells in the ventral and dorsolateral subdomains in unirradiated control mice and at 48 h following 2 Gy. For this analysis, >50 BrdU^+^ cells were scored per mouse from 3 mice per treatment condition. For the irradiated group, this required cell scoring on at least 10 tissue sections per mouse. (G) The representative images for the experiment shown in panel F. Insets magnify cells indicated in the dashed box showing the presence of double-labelled BrdU^+^MAP2^+^ cells at 48 h after 2 Gy. Experiments were carried out on 3-month-old mice, and results represent the mean ± SEM of *n* ≥ 3 mice for each condition. Scale bars, 25 μm. Student *t* test, * *P* < 0.05, ns = not significant. Underlying data can be found in the S1 Data file.

Next, we examined whether the Dcx marker loss represents differentiation of NBs into more mature neurons within the SVZ and looked at the expression of early (neuron-specific class III β-tubulin [Tuj1]) and late (microtubule-associated protein 2 [MAP2]) neuronal differentiation markers in the ventral and dorsolateral regions at 48 h post IR (Fig 4A and 4B). As expected, the percentage of Tuj1^+^ cells decreased after 2 Gy compared with control (Fig 4A–4C) due to its partial expression in NBs, which undergo apoptosis after IR (Fig 2B). The decrease in Tuj1^+^ cells was, however, less than that observed for the Dcx marker (Fig 3B and 3C), suggesting the possibility that early committed neurons are not lost. The overall percentage of mature MAP2^+^ neuronal cells after IR was slightly greater than control, although the difference was not statistically significant (Fig 4B). However, this may be a result of the relatively low number of MAP2^+^ cells arising from differentiation compared to the number already present in the SVZ.

To further examine whether NBs can more rapidly commit to a neuronal fate following IR, we injected BrdU at 4 h and 1 h before IR with 2 Gy and waited 48 h before collection (Fig 4D). The BrdU-labelling analysis, when compared to the data obtained at 6 h (Fig 3E), revealed a decrease in the percentage of BrdU^+^ cells in the ventral subdomain but not the dorsolateral subdomain, consistent with migration of the BrdU^+^ cells towards the rostral migratory stream (Fig 4E). In fact, the percentage decrease in BrdU^+^ cells after IR could predominantly be attributed to loss by apoptosis (approximately 50%). Since the majority of BrdU^+^ cells are Dcx^+^ in unirradiated mice, this raises the possibility that BrdU^+^ cells are retained in the SVZ and undergo differentiation.

To examine this possibility, we co-stained the BrdU labelled cells with Dcx, Tuj1, and MAP2 (Fig 4F). In control mice, the majority of BrdU^+^ cells had a NB phenotype, expressing Dcx and Tuj1, and virtually none of the BrdU^+^ cells co-expressed the more mature MAP2 neuronal marker. Importantly, after IR, a higher percentage of BrdU^+^ cells expressed MAP2 in both the ventral and dorsolateral subdomains, indicating cell cycle exit and differentiation into more mature neurons (Fig 4F and 4G).

Collectively, these findings suggest that loss of the Dcx marker can be attributed to the differentiation of Dcx^+^ cells into mature (MAP2^+^) neurons within the SVZ.

### Progenitors are replenished by qNSC activation

**Fig 5:**
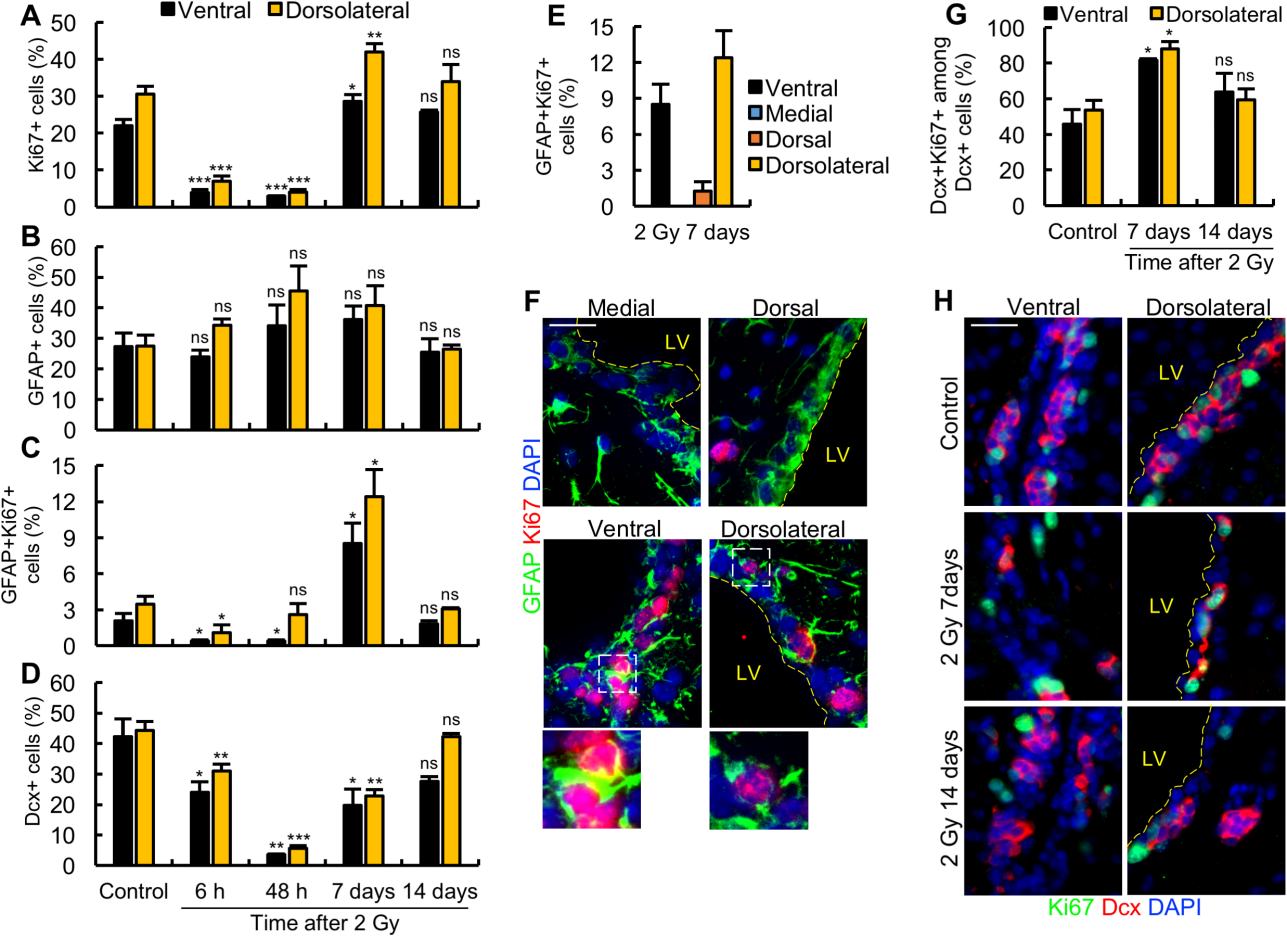
Temporal analysis of the response to 2 Gy within distinct subdomains of the lateral ventricle. (A-D) Adult mice were exposed to 2 Gy and the percentage of Ki67^+^ (panel A), Glial Fibrillary Acidic Protein (GFAP)^+^ (panel B), GFAP^+^Ki67^+^ (panel C), and doublecortin (Dcx^+^) (panel D) cells assessed in the ventral or dorsolateral subdomains as indicated. (E-F) The quantification (panel E) of the percentage of GFAP^+^Ki67^+^ cells in all subdomains and representative images (panel F) at 7 days after 2 Gy. (G-H) The quantification (panel G) of the percentage of Dcx^+^Ki67^+^ cells amongst the Dcx^+^ cells in the ventral and dorsolateral subdomains of unirradiated control mice and at 7 and 14 days post 2 Gy ionising radiation (IR) and representative images (panel H). Experiments were carried out on 3-month-old mice and results represent the mean ± SEM of *n* ≥ 3 mice for each condition. Scale bars, 25 μm. Student *t* test was performed relative to the control group, * *P* < 0.05, ** *P* < 0.01, *** *P* < 0.001, ns = not significant. Underlying data can be found in the S1 Data file.

To assess the longer-term consequences of apoptosis and proliferative arrest, we extended our analysis to 7 and 14 days post IR. First, in the ventral and dorsolateral subdomains, Ki67^+^ cells, which, as described above, were almost undetectable at 6 h and 48 h post IR, increased to levels slightly greater than or similar to that of unirradiated control animals by 7 days (Fig 5A). Thus, either there is a transient (approximately 48 h) arrest of proliferation or a defined mechanism to repopulate the pool of proliferating progenitors. The percentage of GFAP^+^ cells did not change significantly over this time period, although we observed a modest increase at 48 h and 7 days (Fig 5B). Strikingly, GFAP^+^Ki67^+^ (i.e., activated stem cells), which are low in number in control cells, showed a slight increase at 48 h and a >4-fold increase above the control level at 7 days. This was followed by a return to the level seen in unirradiated animals by 14 days (Fig 5C). This demonstrates that exposure to 2 Gy causes marked qNSC activation. Analysis of Dcx^+^ NBs revealed a slower rate of recovery with only half the Dcx^+^ cell numbers being detectable at 7 days and a further increase by 14 days (Fig 5D).

We focused our detailed analysis above on the ventral and dorsolateral subdomains since only these domains contain neural progenitors. Examination of the dorsal and medial regions showed no evidence for NSC activation at 7 days post IR (Fig 5E and 5F), demonstrating that there is no cross talk between the subdomains and that exposure to 2 Gy cannot cause qNSC activation in the medial or dorsal domains. Detailed temporal analysis of the dorsal and medial subdomains for additional markers is shown in S4A–S4D Fig.

These findings reveal that the return of GFAP^+^Ki67^+^ cells precedes the replenishment of Dcx^+^ cells, suggesting that NBs are replenished by activation of qNSCs. This suggestion is further supported by intervening temporal analysis of mice at 3 and 5 days post IR, showing that the increase in GFAP^+^Ki67^+^ cells clearly precedes the return of Dcx^+^ cells (S4C and S4D Fig). Additionally, at 7 days, a larger fraction of Dcx^+^ cells are Ki67^+^, which is consistent with their recovery occurring via stem cell activation (Fig 5G and 5H). Thus, we propose that the major route of Dcx recovery after 2 Gy IR is via qNSC activation.

### IR-induced apoptosis, proliferation arrest, and differentiation are ATM-dependent

**Fig 6:**
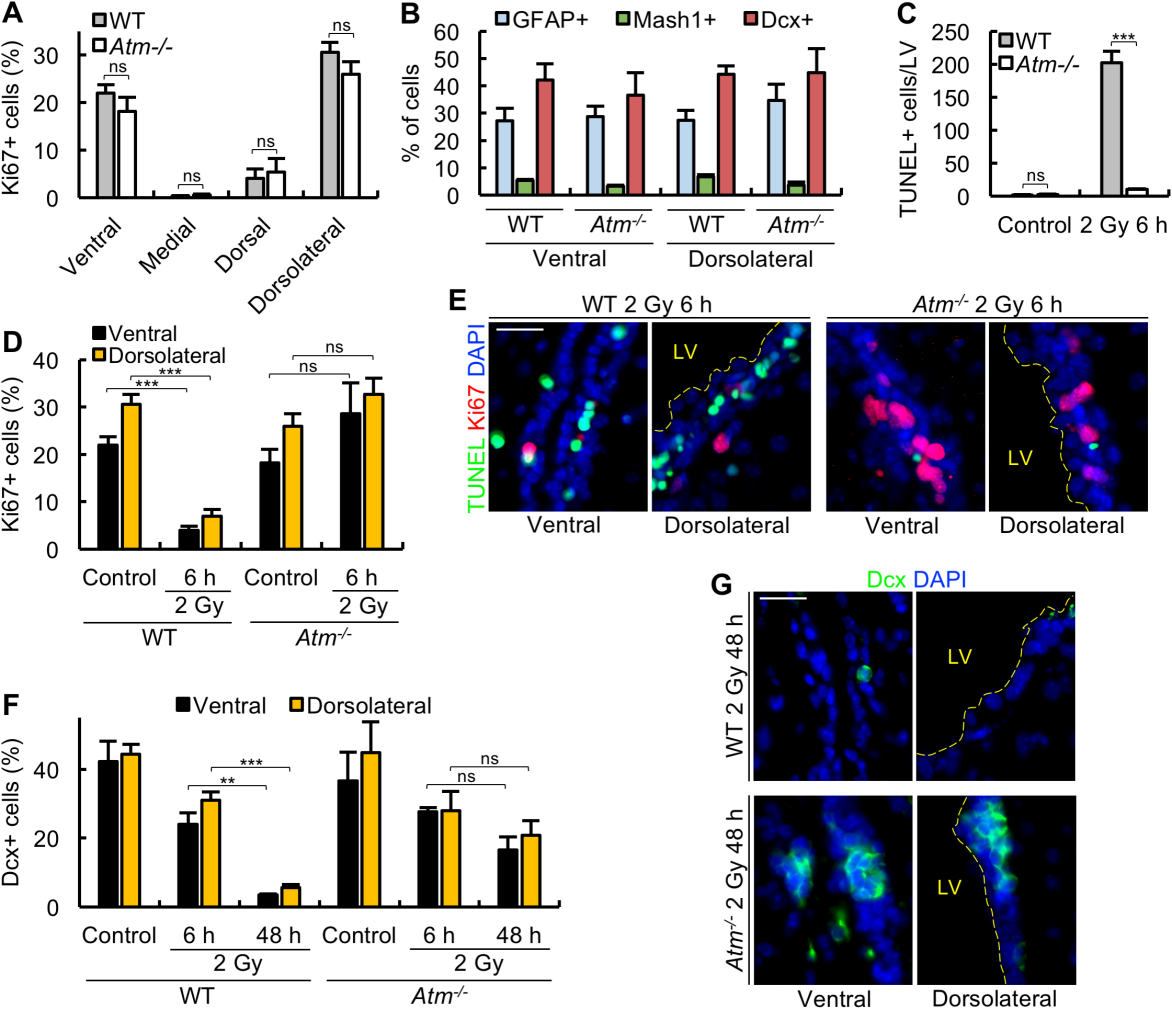
Apoptosis, proliferation arrest, and progenitor marker loss in *Atm*^*-/-*^ mice. (A) *Atm*^*-/-*^ mice show similar levels of proliferating cells in each subdomain to wild-type (WT) mice. The percentage of Ki67^+^ cells was quantified in each subdomain of 3-month-old *Atm*^*-/-*^ mice. Results are compared to age-matched WT mice. (B) *Atm*^*-/-*^ mice show a similar distribution of cell type within the subdomains of the lateral ventricle (LV). (C) *Atm*^*-/-*^ mice fail to activate apoptosis in the LV at 6 h post 2 Gy, as shown by the total number of terminal deoxynucleotidyl transferase-mediated dUTP nick end-labelling (TUNEL)^+^ cells per LV. (D) *Atm*^*-/-*^ mice do not show arrest of proliferation at 6 h post 2 Gy. The percentage of Ki67^+^ cells was quantified for the ventral and dorsolateral subdomains, and data was compared to WT mice. (E) Representative images from experiments shown in panels C and D. (F) *Atm*^*-/-*^ mice do not efficiently lose the Dcx marker post IR. The percentage of Dxc^+^ cells was quantified for the ventral and dorsolateral subdomains at 6 h and 48 h post 2 Gy. (G) Representative images from experiment shown in panel F. A time course analysis to 7 days post IR of Ki67^+^, Dcx^+^, GFAP^+^, and GFAP^+^Ki67^+^ cells in *Atm*^*-/-*^ mice is shown in S5 Fig. The data is discussed in the supplementary figure legend. Experiments were carried out on 3-month-old mice and results represent the mean ± SEM of *n* ≥ 3 mice for each condition. Scale bars, 25 μm. Student *t* test, * *P* < 0.05, ** *P* < 0.01, *** *P* < 0.001, ns = not significant. Underlying data can be found in the S1 Data file.

IR causes double-strand break (DSB) formation and activates ATM-dependent DNA damage-response signalling [23]. However, in cycling cells, ataxia-telangiectasia-related (ATR) can also be activated during replication. In previous studies, we have observed that after 500 mGy, apoptosis in the embryonic ventricular zone (VZ)/SVZ is approximately 70% ATM-dependent, suggesting that ATM is the major activating kinase, but another kinase, most likely ATR, can also contribute [24]. To assess the ATM dependency of the DDR responses in the adult SVZ, we examined apoptosis, proliferation arrest, and Dcx marker loss following exposure to 2 Gy in *Atm*^*-/-*^ mice. First, we examined the distribution of Ki67^+^ cells in the SVZ subdomains and found that, as for control mice, Ki67^+^ cells were predominantly located in the ventral and dorsolateral domains (Fig 6A). The domains also showed a similar distribution of NSCs (GFAP^+^), TAPs (Mash1^+^), and NBs (Dcx^+^) to that of the wild-type (WT) SVZ (Fig 6B). Significantly, we failed to observe either apoptosis or proliferation arrest at 6 h in *Atm*^-/-^ mice (Fig 6C–6E). There was a small reduction of Dcx^+^ cells at 6 h (similar to that observed in WT mice), but, by 48 h, more Dcx^+^ cells remained than observed in WT mice (although slightly lower than at 6 h) (Fig 6F and 6G). Collectively, we conclude that the 3 described DDR responses (apoptosis, Ki67 loss, and differentiation) to 2 Gy IR are ATM-dependent. Further, we did not observe any increase in GFAP^+^Ki67^+^ cells in *Atm*^*-/-*^ mice at 48 h or 7 days post 2 Gy (S5 Fig). These findings are consistent with the notion that a failure to activate the DDR responses precludes qNSC activation. However, despite failing to activate cell death by apoptosis, *Atm*^*-/-*^ cells lack many responses to DSBs and are markedly radiosensitive, undergoing cell death by other processes, such as necrosis, senescence, or ATR-dependent permanent checkpoint arrest. These alternative forms of cell death may also contribute to a failure to activate *Atm*^*-/-*^ qNSCs.

### Resistance to apoptosis is a feature of NSCs unrelated to their quiescence status, repair capacity, or onset of senescence

**Fig 7:**
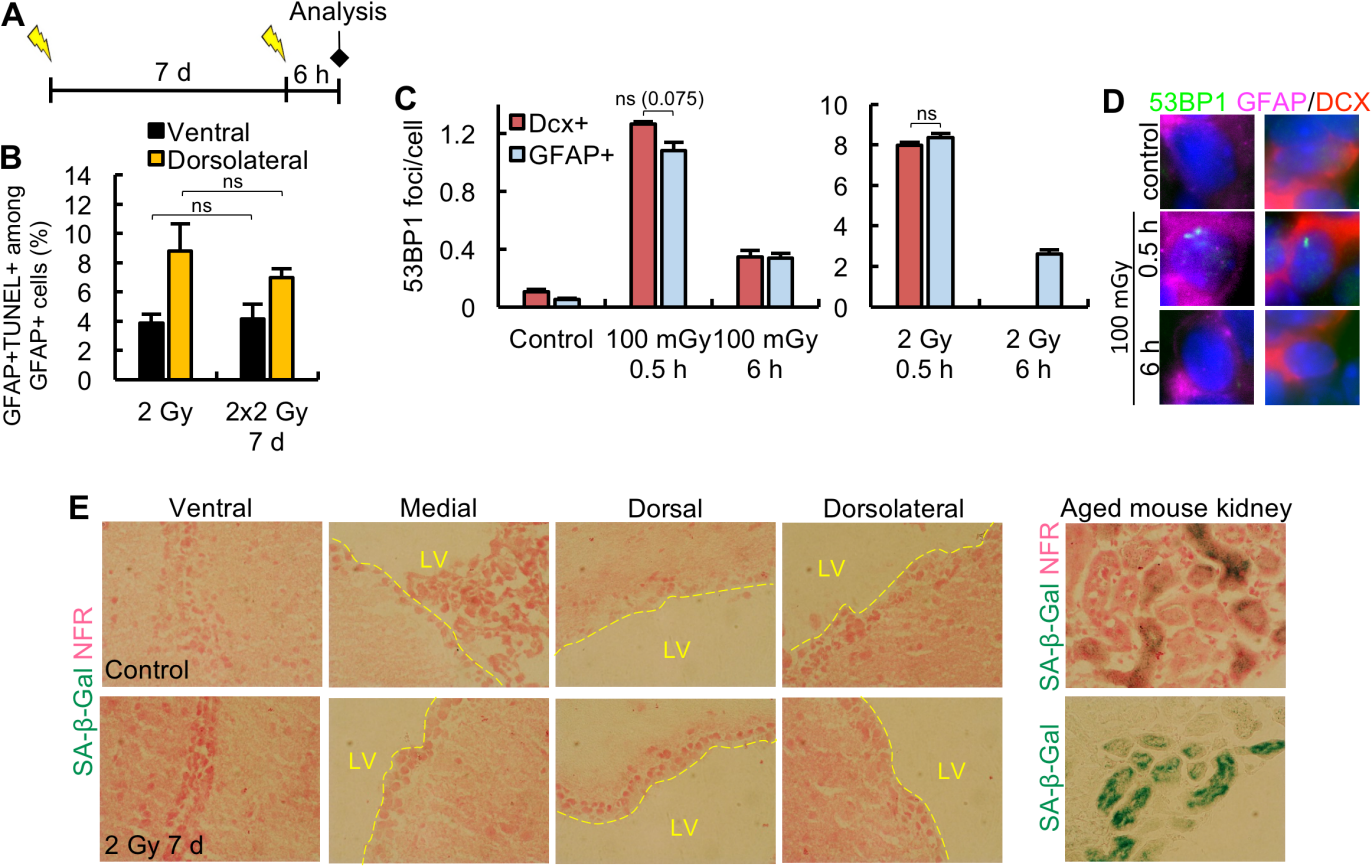
DNA damage responses in the neural stem cells (NSCs). (A-B) Activated Glial Fibrillary Acidic Protein (GFAP)^+^ stem cells do not undergo apoptosis. Panel A shows the protocol for quantifying apoptosis in activated stem cells. In brief, 3-month-old adult mice are exposed to 2 Gy, and, 7 days later, given a second exposure to 2 Gy, then the level of GFAP^+^ terminal deoxynucleotidyl transferase-mediated dUTP nick end-labelling (TUNEL)^+^ cells out of the total GFAP^+^ cells were quantified 6 h later in the ventral (black) or dorsolateral (yellow) subdomains. The level of activated NSCs (GFAP^+^Ki67^+^) was about 4-fold higher at 7 days after the first exposure (Fig 5C), but the level of apoptosis after the second exposure was similar to that observed after a single exposure to 2 Gy. (C-D) Neural stem and progenitor cells show similar levels of double-strand break (DSB) induction and repair. Three-month-old adult mice were exposed to 100 mGy or 2 Gy, and p53-binding protein 1 (53BP1) foci (a DSB marker) were quantified 0.5 h and 6 h later in doublecortin (Dcx^+^) and GFAP^+^ cells. Panel D shows representative images. We have previously observed one-third the level of 53BP1 foci in our tissue analysis compared with that found using cultured fibroblasts [24]. The numbers observed here are consistent with this (approximately 25 foci are observed at 0.5 h post 2 Gy in cultured fibroblasts). The reduced efficiency of DSB detection in vivo could be a consequence of a failure to detect all DSBs throughout the depth of the cell in a tissue section. (E) Stem cells do not show senescence at 7 days post 2 Gy. Senescence was assessed using β-galactosidase (SA-β-Gal) and nuclear fast red (NFR) as counterstain in individual subdomains of the lateral ventricle (LV). SA-β-Gal^+^ cells were observed endogenously in the kidney of aged (21-month-old) mice. For quantification, *n* = 2 mice for each condition were analysed. Experiments in A-D were carried out on 3-month-old mice, and results represent the mean ± SEM of *n* ≥ 3 mice for each condition. Student *t* test, ns = not significant. Underlying data can be found in the S1 Data file.

The analysis of Mash1^+^ and Dcx^+^ cells demonstrates that sensitivity to apoptosis after 2 Gy is a feature of these neural progenitors rather than being a direct consequence of their proliferative status (Fig 2C and 2D). We, therefore, sought to investigate whether, conversely, the resistance of GFAP^+^ cells to apoptosis is a consequence of their quiescent status. To address this, we examined whether activated GFAP^+^Ki67^+^ cells undergo apoptosis. It was difficult to address this after a single dose of 2 Gy because the number of GFAP^+^Ki67^+^ cells is very low (approximately 10% of GFAP^+^ cells), and the Ki67 marker is not expressed at 6 h after 2 Gy, the optimal time for assessing apoptosis. We, therefore, took an alternative approach to address this question by exploiting the marked increase in activated NSCs (i.e., GFAP^+^Ki67^+^) at 7 days post 2 Gy IR. Three-month-old mice were exposed to 2 Gy IR, then 7 days later exposed to a second dose of 2 Gy, and GFAP^+^TUNEL^+^ cells quantified 6 h later (Fig 7A). The magnitude of apoptosis was compared to that observed in NSCs at 6 h post a single dose of 2 Gy (Fig 7B). Strikingly, despite a 4-fold increase in activated GFAP^+^ cells at 7 days post 2 Gy (Fig 5C), the second exposure to IR did not promote any increase in apoptosis amongst GFAP^+^ cells in the ventral or dorsolateral domain. This experiment provides strong evidence that activated NSCs, like their quiescent counterpart, are resistant to IR-induced apoptosis, consolidating the notion that sensitivity (or resistance) to apoptosis is a feature of the cell type rather than an indirect consequence of proliferation.

It has also been proposed that hair follicle BSCs have an upregulated capacity to repair DSBs, which is causally related to their resistance to apoptosis [8]. To gain insight into the basis underlying the resistance of NSCs and sensitivity of the progenitors to apoptosis, we examined whether NSCs in the SVZ incur the same DSB induction level as Dcx^+^ NBs and whether they display any changes in DSB repair capacity. To assess this, adult mice were exposed to 100 mGy or 2 Gy and p53-binding protein 1 (53BP1) foci, a marker of DSB formation, quantified 0.5 h and 6 h later in GFAP^+^ and Dcx^+^ cells. A dose of 100 mGy was used since this induces <1% apoptosis in progenitors, allowing foci numbers to be enumerated in predominantly nonapoptotic cells. Although 100 mGy only induces 1 to 2 foci/cell, foci numbers could be accurately assessed by scoring a large area and multiple tissue sections. Repair was not assessed in Dcx^+^ cells after 2 Gy due to presence of apoptotic cells. We observed similar levels of induced DSBs in Dcx^+^ and GFAP^+^ cells (Fig 7C and 7D). The slightly greater levels in the Dcx^+^ cells after 100 mGy is not significant (*P* = 0.075) and insufficient to account for the marked distinction in apoptotic sensitivity and was not observed after 2 Gy. Assessment of foci numbers at 6 h post 100 mGy showed a similar level of residual DSBs in Dcx^+^ and GFAP^+^ cells, suggesting a similar DSB repair capacity, at least over this time scale (Fig 7C and 7D).

Finally, we also examined whether NSCs undergo senescence rather than apoptosis using β-galactosidase (SA-β-gal) as a marker of senescent cells. Whilst we were able to detect the senescence marker, SA-β-gal, in the kidney of aged 21-month-old mice, we did not detect any SA-β-gal^+^ cells in any of the SVZ subdomains at 7 days post 2 Gy (Fig 7E).

Thus, although limited by the restrictions of in vivo analysis, we have not found any evidence that the resistance of NSCs to apoptosis is a direct consequence of a protective environment, quiescence, or an enhanced DSB repair capacity. Additionally, we could not detect any senescence at 7 days post 2 Gy in the SVZ subdomains.

### The neonatal and adult SVZ show similar sensitivity to IR-induced apoptosis

**Fig 8:**
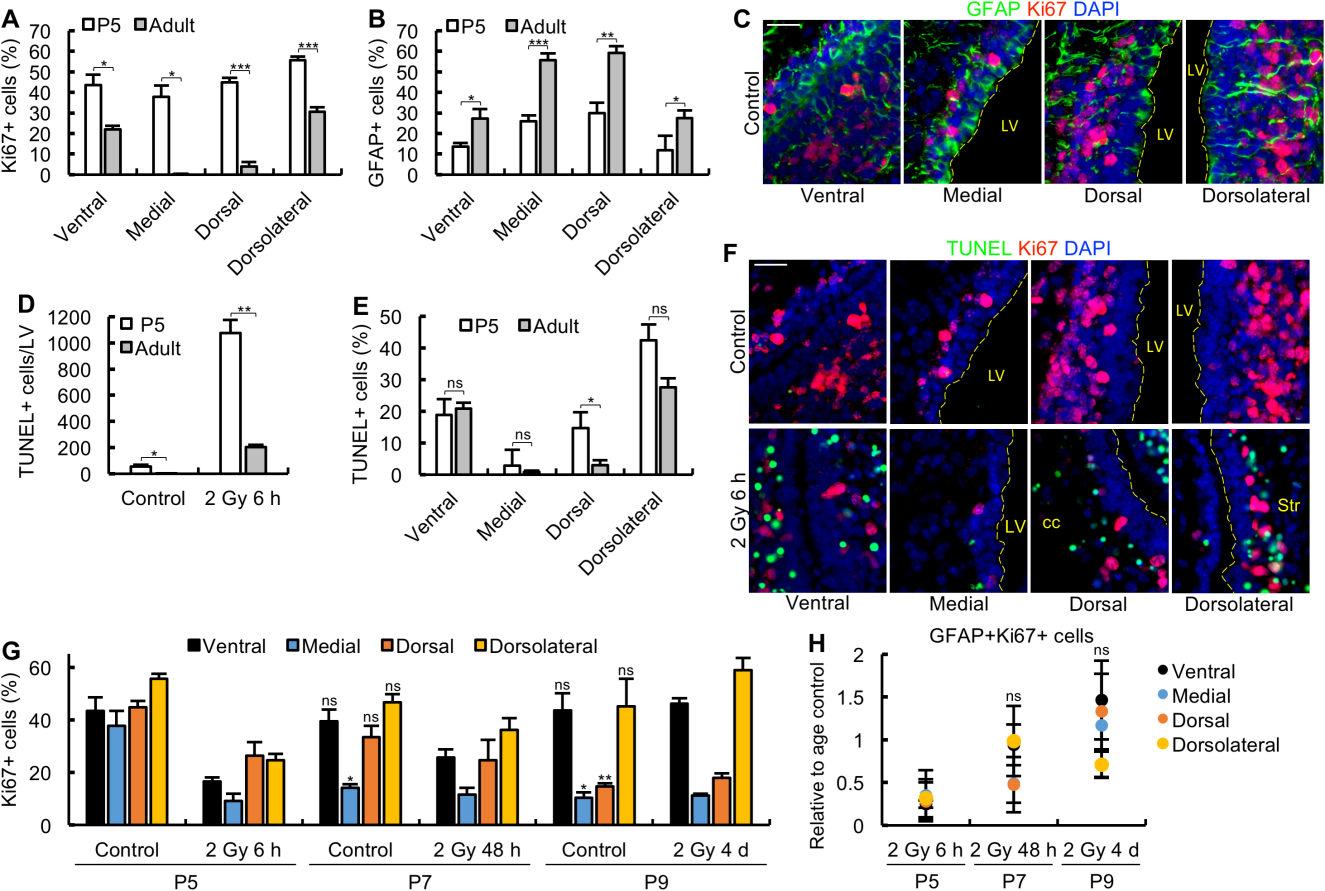
Neonatal mice do not efficiently arrest proliferation after 2 Gy. (A-C) Neonatal (P5) mice show enhanced proliferative cells and reduced Glial Fibrillary Acidic Protein (GFAP)^+^ cells in each subdomain relative to adult mice. The percentage of Ki67^+^ cells (panel A) and GFAP^+^ cells (panel B) in each lateral ventricle subdomain were quantified in P5 and adult mice. Panel C shows representative images of control mice at P5. (D) The level of apoptosis per lateral ventricle (LV) is enhanced in P5 compared to adult mice. (E) The level of apoptosis per cell is similar in the ventral and dorsolateral subdomains in P5 and adult mice. Apoptosis is assessed as the percentage of terminal deoxynucleotidyl transferase-mediated dUTP nick end-labelling (TUNEL)^+^ cells out of the total 4′,6-diamidino-2-phenylindole (DAPI)^+^ cells in each subdomain. (F) Representative images of P5 mice from the experiment shown in panel E. (G) Proliferation is not efficiently arrested in P5 mice post 2 Gy. The percentage of Ki67^+^ cells were quantified in each subdomain at P5, P7, and P9 mice either with or without exposure to 2 Gy on P5 as indicated. P5, P7, and P9 respectively correspond to 6 h, 48 h, and 4 days post irradiation at P5. In panel G, Student *t* tests were performed relative to the control group at P5. (H) The level of GFAP^+^Ki67^+^ cells out of the total GFAP^+^ cells were assessed at the specified times post 2 Gy in each subdomain as indicated. The results and Student *t* tests are expressed relative to the level in untreated mice of the same age. Experiments were carried out on P5, P7, and P9 mice, and results represent the mean ± SEM of *n* ≥ 3 mice for each condition. cc, corpus callosum. Str, striatum. Scale bars, 25 μm. Student *t* test, * *P* < 0.05, ** *P* < 0.01, *** *P* < 0.001, ns = not significant. Panels C and F were originated from 4-channel images. Underlying data can be found in the S1 Data file.

The embryonic VZ/SVZ represents the predominant forebrain region in the embryo and undergoes rapid proliferation from E11.5 to E16.5. Although the size of the neonatal SVZ and the extent of proliferation diminishes post birth, both are greater at P5 than observed in the adult SVZ [17]. Slowly dividing qNSCs increase in level and adopt an astrocytic morphology during the early neonatal stage with the extent of proliferation and the number of progenitors decreasing [17]. Whether the distinct subdomains differ in the rate at which these changes arise has not been examined. First, we assessed the percentage of Ki67^+^ and GFAP^+^ cells in the distinct subdomains of P5 mice (Fig 8A and 8C). Since the SVZ is larger in neonatal (P5) mice compared to 3-month-old mice, we expressed the results as a percentage of cells within each domain. These findings reveal, as expected, that there is an increased level of proliferative cells in all subdomains of P5 mice compared to adult mice; the increase is approximately 2-fold in the ventral and dorsolateral regions, in which there are detectable Ki67^+^ cells in the adult; a more dramatic increase is observed in the medial and dorsal regions, in which few proliferative cells are observed in the adult (Fig 8A). The number of GFAP^+^ cells in each subdomain showed the converse picture, that is, a lower number of GFAP^+^ cells (Fig 8B). Quantification of apoptosis revealed approximately 5-fold increased apoptosis in the entire SVZ, most likely a consequence of the increased size of the SVZ as well as the increased level of apoptotic-sensitive progenitor cells (Fig 8D). However, when assessed as the percentage of cells in each subdomain, a similar level of apoptosis to that found in the adult was observed in the ventral and dorsolateral regions. Since the number of Ki67^+^ cells was slightly enhanced in these 2 subdomains, this suggests that there is a similar or possibly slightly reduced sensitivity for each progenitor cell to undergo apoptosis at P5 (Fig 8E and 8F). Additionally, apoptosis was enhanced in the dorsal region and slightly in the medial region due to the presence of proliferating cells at this stage (Fig 8E).

Collectively, these findings suggest that although the overall level of apoptosis in the neonatal SVZ is greater than in the adult SVZ, this is largely attributable to the increased size of the neonatal SVZ. When evaluated on a per cell basis, the sensitivity of each progenitor cell to activate apoptosis appears similar to that in the adult SVZ.

### Proliferation is not efficiently arrested in the neonatal SVZ after 2 Gy IR

In adult mice, we observed a marked decrease (5- to 6-fold) in Ki67^+^ cells in the ventral and dorsolateral subdomains at 6 h post 2 Gy IR, which occurs in most progenitor cells independently of whether they succumb to apoptosis (Fig 3B and 3C). We, therefore, examined whether the Ki67 marker is similarly lost following exposure of P5 mice to 2 Gy. Strikingly, at 6 h post 2 Gy, there was a much smaller reduction (2-fold) in the ventral, dorsolateral, and dorsal subdomains of P5 mice (Fig 8G). The reduction in Ki67^+^ cells in the medial region was more dramatic, but nonetheless, cells expressing the Ki67 marker were readily detectable. This difference between adult and neonatal mice appears to represent a distinction in the percentage of cells arresting proliferation rather than simply being a consequence of the increased number of Ki67^+^ cells in the neonatal SVZ.

In adult mice, the marked reduction in Ki67^+^ cells remained at 48 h post IR (Fig 3B and 3C). To assess the response at 48 h in the neonates, it was necessary to estimate the percentage of Ki67^+^ cells in unirradiated P7 mice (i.e., the equivalent age of mice exposed at P5 and assessed 48 h post IR) since there are dynamic changes in proliferative capacity during this early neonatal period. This analysis revealed that the number of Ki67^+^ cells in the medial region in unirradiated P7 neonates is substantially lower than in P5 neonates, suggesting that the proliferative capacity of this subdomain diminishes (i.e., “ages”) more rapidly than the other subdomains (Fig 8G). The number of Ki67^+^ cells was similar in the unirradiated control or irradiated medial subdomain at P7, suggesting that the decrease with age is not additive with any radiation-induced decrease. In the other subdomains, the percentage of Ki67^+^ cells was similar between P5 and P7 unirradiated control neonates (Fig 8G). Following IR at P5, there was a small increase in Ki67^+^ cells at 48 h compared to the level of 6 h, suggesting that replenishment was already commencing. At P9, the distribution of Ki67^+^ cells within the subdomains of unirradiated mice resembled that of adult mice, although the percentage of proliferating cells was nearly 2-fold greater than in the adult mice. At 4 days following exposure of P5 mice (i.e., P9), the number of Ki67^+^ cells in each subdomain was similar to unirradiated P9 mice and greater than at 6 h post IR. Thirty percent of the neonatal SVZ cells undergo apoptosis by 6 h (Fig 8E), suggesting that all the remaining cells (active NSCs and/or progenitors) express the Ki67 marker by 48 h (Fig 8G). We consider it unlikely that these cells were recovered by qNSC activation since this takes longer than 48 h in the adult SVZ (Fig 5C). To substantiate this, we quantified the number of GFAP^+^Ki67^+^ cells following IR exposure of P5, P7, and P9 mice exposed or not to IR at P5 (Fig 8H). At 6 h, the number of GFAP^+^Ki67^+^ cells was reduced 2- to 4-fold relative to unirradiated neonatal mice similar to that observed for all Ki67^+^ cells (which predominantly reflect progenitor cells and not NSCs). By 48 h and 4 days post IR, the level of GFAP^+^Ki67^+^ NSCs had increased to that observed in unirradiated neonates, but the rate of increase paralleled the response of all Ki67^+^ cells. We conclude that the kinetics of proliferating progenitor cell recovery strongly suggests that it occurs from surviving irradiated progenitors rather than via the activation of qNSCs as in adult mice.

Collectively, these findings reveal important distinctions between the DDR to 2 Gy IR in the neonatal compared to the adult SVZ. Although the ability of progenitor cells to activate apoptosis in neonates is similar to adults with substantially more apoptosis taking place due to the enhanced size of the SVZ and increased number of progenitors, the neonatal SVZ fails to efficiently arrest proliferation, and the recovery of proliferating progenitors occurs more rapidly. The kinetics of recovery and analysis of activated NSCs, moreover, strongly suggests that in neonatal mice the progenitors repopulate from inefficiently arrested irradiated progenitors rather than via qNSC activation.

## Discussion

### Neural progenitors in the adult SVZ undergo apoptosis; NSCs are resistant

In this study, we examine the response to DNA damage in the SVZ exploiting recent understanding of its spatial organization. This revealed distinct responses of the neural stem and progenitor cells and insight into the process leading to qNSC activation. Additionally, a temporal analysis has shown distinctions in the responses of the adult versus the juvenile SVZ to DNA damage, which is of significance given their differing sensitivities to carcinogenesis. First, we show that NSCs are resistant to apoptosis, whilst TAPs and NBs activate apoptosis in a dose-dependent manner. This parallels the response of HSCs and mammary stem cells and their respective progenitors [7]. HSCs and NSCs have distinct turnover rates, suggesting that the apoptotic sensitivity is not unique to more proliferative stem cell compartments [25]. The apoptotic sensitivity of progenitors is not a direct consequence of replication but rather represents a programmed cell type–specific response.

### Two novel ATM-dependent DDR responses in the SVZ: Proliferative arrest and differentiation

We report 2 additional novel responses to exposure to 2 Gy IR: (1) rapid (by 6 h) proliferation arrest, measured by Ki67 and BrdU/EdU incorporation, and (2) loss (by 48 h) of cells bearing the progenitor marker, Dcx. Cell cycle checkpoint arrest is a well-characterized response of cultured cells to IR and has also been observed in tissues [24,26]. However, it is unclear if the Ki67 marker loss and lack of EdU incorporation observed in the adult SVZ after 2 Gy represents a consequence of checkpoint arrest or a distinct response. In the SVZ, after 2 Gy, the Ki67 marker loss appears to be sustained for at least 48 h, with recovery arising from activated NSCs rather than from preexisting progenitor cells. In contrast, after 2 Gy G1/S or G2/M checkpoint arrest in cultured primary cells is more transient [27]. We provide evidence that the second response observed, namely loss of Dcx^+^ progenitors, can be attributed to enhanced differentiation of immature into mature neurons. One study using cultured cells observed that DNA damage can induce astrocytic differentiation in NSCs derived from embryonic stem cells [28]. Additionally, DNA damage has been shown to induce terminal differentiation in HSCs [9,10]. Further studies are required to understand the basis underlying this important observation. Importantly, apoptosis, proliferative arrest, and differentiation are ATM-dependent, suggesting that they represent a coordinated response to DNA damage in the adult neural stem cell compartment. These responses appear linked to the activation of qNSCs since NSC activation is only observed in the subdomains where these responses are seen. Further, qNSC activation is not observed in *Atm*^*-/-*^ mice. However, we resist causally linking these endpoints since ATM loss, despite overriding cell death by apoptosis, can lead to dramatic radiation sensitivity (via cell death by other processes), rendering *Atm*^*-/-*^ qNSCs unable to become activated.

### Why do progenitor cells undergo apoptosis?

Other studies describing the resistance of quiescent stem cells to apoptosis frequently describe them as being radioresistant [29]. However, apoptosis represents a form of cell death that can be sensitively monitored, and the absence of apoptosis should not be taken to assume radioresistance. Cultured primary fibroblasts, for example, do not undergo IR-induced apoptosis, yet they lose proliferative capacity due to lethal chromosome breaks or aberrations [30]. Although there are differences in the precise shape of the survival curves, assessing survival of NBs by the percentage of cells without apoptosis shows a similar survival response to analysis of radiosensitivity of cultured fibroblasts via clonogenic survival assays (S6A Fig). Furthermore, the level of DNA damage incurred and NHEJ repair appears similar between qNSCs and progenitors. Thus, as a working hypothesis, we propose that progenitors undergo apoptosis, a form of cell death that causes rapid cell loss, to promote recovery via quiescent stem cell activation. Two additional responses, namely proliferation arrest and progenitor cell differentiation potentially serve to enhance quiescent stem cell activation. These ATM-regulated responses have the important consequence of changing the balance of signals that confer homeostasis between stem cell quiescence/activation causing activation of qNSCs. Enhancing differentiation may function to diminish progenitor cells, thereby increasing the signal for qNSC activation. Additionally, progression through replication or mitosis in the presence of DNA damage can cause lethality or enhance misrepair. Although cells activate DDR checkpoints, they have limitations and, as evident from the studies on potentially lethal damage repair (PLDR), cycling cells have reduced survival and chromosome rearrangements relative to cells that have exited the cell cycle [27]. Thus, activating responses to restrict the recovery of irradiated progenitors, the cells most vulnerable to IR-induced rearrangements via their proliferative capacity, will have the additional advantage of restricting carcinogenesis. Instead, promoting recovery via the activation of a quiescent population will, as demonstrated by many classic radiobiology studies, enhance survival and, importantly, reduce translocations, despite the employment of NHEJ [30]. In summary, we propose that apoptosis is not so much a form of sensitive cell death but rather a form of cell death that causes rapid cell loss in the germinal centre, thereby promoting quiescent stem cell activation. The processes of proliferation arrest and differentiation represent complementary components of this DDR. The ability of DNA damage to promote exit from dormancy in HSCs has also recently been demonstrated, and, here, we link this to cell loss by apoptosis [6]. Significantly, we do not observe qNSC activation in the medial or dorsal regions, which do not undergo apoptosis.

### Distinct responses of adult versus neonatal mice to IR exposure

Radiation exposure of the neonatal brain has been shown to diminish cognitive function and to enhance carcinogenesis [20–22,31]. Our subdomain analysis substantiates other studies showing that the juvenile SVZ has a larger number of proliferating progenitors than the adult brain and, as a corollary, fewer qNSCs [32]. Although this results in an increased number of apoptotic-sensitive progenitors, their sensitivity appears similar to that of adult progenitors. Consequently, overall there is a greater level of apoptosis in the neonatal compared to the adult SVZ, which likely reflects the enhanced sensitivity of the neonatal brain to IR. Strikingly, however, the neonatal progenitor cells have a diminished ability to undergo proliferative arrest at P5 compared to adult progenitors and, moreover, recover proliferative capacity more readily. Indeed, the marked difference in the kinetics of recovery of proliferation strongly suggests that the irradiated progenitors regain proliferative capacity after a brief arrest rather than repopulation arising as a consequence of quiescent stem cell activation. Whilst exposure of the adult brain to 2 Gy appears to promote the repopulation of NBs from qNSCs, the progenitors are rapidly replenished in neonates via a mechanism that does not require extensive NSC activation (shown in S6B Fig). Thus, the NBs are derived from irradiated proliferating cells in neonates but from activated NSCs in the adult mice, a distinction which may influence the level of rearrangements they contain, and hence their ability to become carcinogenic. These findings also suggest that after 2 Gy IR, the activation of apoptosis is not the sole factor promoting qNSC activation but involves, additionally, proliferative arrest and differentiation. We note also that in this study we have focused on the response to 2 Gy IR. However, such a dose is rarely encountered outside a radiotherapy setting, and it will be important to assess these responses using much lower, more physiological doses. More studies are needed to determine whether similar responses arise in other stem cell compartments.

### Subdomain analysis

Central to our analysis is the exploitation of recent insight into the subdomain structure of the SVZ around the lateral ventricle [17,18,33]. Indeed, it is our combined spatial and temporal analysis which has provided novel insight into the DDR in the SVZ. Importantly, in the context of adult neurogenesis and the DDR, our findings show that the distinct subdomains differ in their degree of “ageing”, which is detectable both from the adult and neonatal analysis. The medial region appears to “age” and lose proliferative capacity most rapidly, followed by the dorsal region. The ventral and dorsolateral regions maintain some neurogenic capacity at least to 3 months of age. This feature lies at the basis of the difference in the DDR between the subdomains. A consequence is that the neuronal subtypes derived from each subdomain will differ in their renewal capacity with age and, of relevance here, differ in their response to DNA damage.

In summary, by exploiting spatial analysis of subdomains within the SVZ, we show that NSCs are resistant to radiation-induced apoptosis, whilst the progenitors, TAPs and NBs, succumb to apoptosis. This response is not a direct consequence of progenitor cell proliferation but reflects a programmed cell type response. Additionally, in the adult SVZ, the proliferation and progenitor markers are lost within 6 h to 48 h post IR in an ATM-dependent manner. This drives activation of qNSCs, which repopulate the progenitor cell pool by 14 days post exposure (see S6B Fig for a model). Temporal analysis of this response reveals that the progenitor cells in the neonatal SVZ similarly undergo apoptosis but display a milder decrease in the proliferation marker and more rapidly repopulate the progenitor pool. Collectively, the findings suggest that after 2 Gy adult mice replenish progenitor cells by qNSC activation, whilst the progenitor cells in neonatal mice that do not succumb to apoptosis can rapidly resume proliferation. We propose that apoptosis is not so much a sensitive form of cell death but rather represents a form of cell death that encourages quiescent stem cell activation, and that this response is additionally promoted by arrest of progenitor proliferation and marker loss, likely reflecting differentiation.

## Materials and methods

### Mice

C57BL/6 and *Atm*^*-/-*^ (129/SV x C57BL/6) mice were used in this study. *Atm*^*-/-*^ mice were generated and genotyped as described previously [34,35]. The day of birth was designated as postnatal day 0 (P0). Neonatal mice were used at P5, P7, P9, and P12. Adult mice were 3-month-old. Untreated age-matched mice were used as control. All animal experiments were performed in accordance with accepted standards of animal welfare approved by the United Kingdom Home Office and complied with the Animals (Scientific Procedures) Act 1986.

### Irradiation treatment

Irradiation was performed using an HS X-ray system (A.G.O. installation, UK). Mice were exposed to a total-body dose of either 100 mGy or 2 Gy (dose rate of 0.5 Gy/min at 250 kV potential).

### Topographical division

Coronal sections of the adult and neonatal brain were taken to correspond to where the lateral ventricle (LV) is maximally extended along the dorsal-ventral axis at plane positions relative to bregma between +1.2 and +0 mm (brain atlas: Paxinos and Franklin, 2008). The SVZ was topographically divided into 4 subdomains: ventral, the area delineating the lower ventral one-third of the LV in correspondence of the nucleus accumbens; medial, the area corresponding to the middle-upper medial wall of the LV in correspondence of the caudal part of the lateral septal nucleus; dorsal, the area adjacent to the corpus callosum encompassing the dorsal roof of the LV; and dorsolateral, the upper dorsal one-third of the caudoputamen [16,33].

### Immunofluorescence

Brains were first fixed with 4% paraformaldehyde (PFA) (Santa Cruz Biotecnology, cat# SC-281692), cryoprotected in 25% sucrose/PBS solution, and frozen after OCT embedment (Thermo Fisher Scientific, cat# 12678646). Samples were cryosectioned (coronal view, 7 μm) on a CM1900 cryostat (Leica) and mounted on Superfrost slides (Thermo Fisher Scientific, cat# 10149870). Antigen retrieval was performed according to the manufacturer’s instructions (Histo VT One, Fine Chemicals Products Ltd, cat# 06380–05). Slides were then washed 3 times with PBS and blocked in 5% serum (donkey or goat), 1% bovine serum albumin (BSA), and 0.4% Triton X-100 in PBS for 1 h at room temperature (RT). Primary antibody incubation was done in a humidified chamber overnight at RT. The following antibodies were used in this study: 53BP1 (rabbit, 1:500, Bethyl Laboratories, cat# A300-272A), Cleaved Caspase-3 (rabbit, 1:200, Cell Signaling, cat# 9579S), Doublecortin (goat, 1:400, Santa Cruz Biotechnology, cat# sc-8066), GFAP (rabbit, 1:500, Abcam, cat# ab7260; mouse, 1:400, Millipore, cat# MAB360), Ki67 (rat, 1:500, eBioscience, cat# 14–5698), Mash1 (rat, 1:100, R&D Systems, cat# MAB2567; mouse, 1:100, BD Biosciences, cat# 556604), Tuj1 (mouse, 1:400, Sigma-Aldrich, cat# T8578; rabbit, 1:100, Sigma-Aldrich, cat# T2200), MAP2 (rabbit, 1:400, Millipore, cat# AB5622), Iba1 (rabbit, 1:1000, Wako Chemicals USA, cat# 019–19741), Sox2 (mouse, 1:100, Cell Signaling, cat# 4900), and Nestin (mouse, 1:200, Millipore, cat# MAB353).

Slides were washed 3 times with PBS and then incubated with the relative Alexa Fluor 488, 555, 594, and 647 conjugated secondary antibodies for 1 h at RT (host either donkey or goat, 1:500, Thermo Fisher Scientific). Sections were counterstained with DAPI and mounted with Vectashield (Vector Laboratories Ltd, cat# H-1000).

### BrdU and EdU in vivo labelling

BrdU (Sigma-Aldrich, cat# B5002) was injected intraperitoneally at 50 mg/kg body weight at 4 h and 1 h before irradiation. EdU (Thermo Fisher Scientific, cat# A10044) was injected intraperitoneally at 50 mg/kg body weight at 1 h before the mice were killed. For BrdU detection, sections were denatured in 2.5 M HCl for 25 min at RT, washed with PBS, and subjected to standard immunofluorescence staining using antibodies against BrdU (MoBU-1) (1:100, mouse, Thermo Fisher Scientific, cat# B35128) and BrdU (BU1/75) (1:100, rat, Biorad, cat# OBT0030CX). EdU incorporation was measured using the Click-iT EdU Alexa Fluor 647 imaging kit (Thermo Fisher Scientific, cat# C10340).

### TUNEL staining

TUNEL staining was performed after immunofluorescence staining according to the manufacturer’s instructions (Roche, cat# 11684795910).

### Senescence β-galactosidase staining

Brains were embedded in OCT and flesh frozen in liquid nitrogen after resection. Frozen tissues were immediately sectioned (coronal, 4 μm) and mounted onto Superfrost slides.

Cellular senescence was assessed using the Senescence β-Galactosidase Staining Kit (Cell Signaling, cat# 9860) according to the manufacturer’s instructions and counterstained with Nuclear Fast Red (Vector Laboratories, cat# H-3403).

### Imaging and quantification

Olympus IX73 and Carl Zeiss microscopes were used for immunofluorescence imaging with 40x and 100x objectives using Micro-Manager software. Bright-field imaging was performed using a Zeiss Axiovert microscope. Image processing and analysis was performed using ImageJ (NIH). Between 3 to 6 tissue sections were analysed for each individual mouse. Quantification of marker-positive cells by immunofluorescence was carried out in at least 200 cells for each SVZ subdomain per section (with a minimum of 600 cells/subdomain scored per mouse). Quantification of 53BP1 foci was done by scrolling through the entire section depth with a 100x objective in at least 100 cells per section.

### Statistics

Values represent the mean and standard error of at least 3 independent experiments. Unpaired Student *t* tests were used for the determination of *P* values.

We used between 3 to 6 mice per each experimental group to allow basic statistical inference given a significant *P* value of 0.05.

## Supporting information

S1 Fig Details of the sub-domains within the lateral ventricle. A. Schematic representation of the olfactory bulb (OB) neurons according to their spatial origin. Red, blue and green denote neurons that have originated from NSCs located in the dorsal, medial and ventral/dorsolateral sub-domains of the SVZ respectively. The medial wall predominantly generates calretin-expressing (CR^+^) periglomerular cells (located in the superficial layers of the OB). The ventral/dorsolateral wall produces largely deep granule cells (located in the deep layers of the OB). Whereas the dorsal wall gives rise to tyrosine hydroxylase-expressing (TH^+^) periglomerular and superficial granule cells in addition to a small population of juxtaglomerular glutamatergic neurons [17]. B. TUNEL staining of the isocortex in adult mice without IR or 6 h post 2 Gy. No TUNEL^+^ cells could be detected in the isocortex after IR demonstrating that the differentiated isocortex is resistant to apoptosis. Scale bars, 100 μm. C. Distribution of Ki67^+^ cells in the ventral and dorsolateral sub-domains of the lateral ventricle. D. Distribution of TUNEL^+^ cells in the ventral and dorsolateral sub-domains of the lateral ventricle. E. Separate color channels of the images presented in Fig 1D showing TUNEL^+^ cells (green), Ki67^+^ cells (red), GFAP^+^ cells (magenta), and DAPI staining (grey). Scale bars, 25 μm. Underlying data can be found in the S1 Data file.
(TIF)

S2 Fig Details of the response of the adult SVZ to 2 Gy IR. A. Quantification of the total number of TUNEL^+^ and Casp3^+^ cells per lateral ventricle (LV) at 6 h post 2 Gy. B. The percentage of Casp3^+^BrdU^+^ out of the total Casp3^+^ cells for the experiment depicted in Fig 2C. C. Quantification of DAPI^+^ cells per area in the sub-domains of the SVZ of untreated control mice and irradiated mice at 48 h following 2 Gy IR. D. Thickness of the SVZ walls in the sub-domains analysed in S2C Fig. Black, ventral; blue, medial; orange, dorsal; yellow, dorsolateral. Student’s t-test, ns = not significant. Underlying data can be found in the S1 Data file.
(TIF)

S3 Fig Microglial Iba1 expression at 48 h post 2 Gy IR. A. Quantification of the total number of Iba1^+^ cells per lateral ventricle (LV) of control and irradiated mice at 48 h post 2 Gy. B. Percentages of Iba1^+^ cells in the ventral and dorsolateral sub-domains of the SVZ as indicated above. C. Representative images of Iba1 staining from experiment shown in panel B. D. Percentages of Iba1^+^ cells in the differentiated isocortex (CTX) of control and irradiated mice at 48 h post 2 Gy. E. Representative images of experiments carried out in panel D. Experiments were carried out on 3 month old mice and results represent the mean ± SEM of n ≥ 3 mice for each condition. Scale bars, 25 μm. Student’s t-test, ns = not significant. Underlying data can be found in the S1 Data file.
(TIF)

S4 Fig Temporal analysis of all sub-domains up to 14 days post IR. Analysis of all sub-domains for the data shown in Fig 5. In addition, data is shown for analysis of mice at 3 and 5 days post IR. Only a single mouse was quantified for each of these two time points (hence error bars are not included). The data shows quantification of (A) Ki67^+^ cells, (B) GFAP^+^ cells, (C) GFAP^+^Ki67^+^cells, and (D) Dcx^+^ cells. Underlying data can be found in the S1 Data file.
(TIF)

S5 Fig Additional analysis of the SVZ in *Atm^-/-^* mice following 2 Gy IR. A. The percentage of Ki67^+^ cells in the ventral and dorsolateral domains up to 7 days post exposure to 2 Gy in *Atm^-/-^* mice. For comparison the response of WT mice is shown in Fig 5A. Notably, the number of Ki67^+^ cells is not reduced at 6 h post IR. By 48 h the level of Ki67^+^ cells is slightly reduced but remains higher than in WT mice at 48 h. Further, the number of Ki67^+^ cells at 7 days post IR does not increase. B. Change in the percentage of GFAP^+^ cells in the ventral and dorsolateral domains up to 7 days post exposure to 2 Gy in *Atm^-/-^* mice. C. Change in the percentage of Dcx^+^ cells in the ventral and dorsolateral domains up to 7 days post exposure to 2 Gy in *Atm^-/-^* mice. Similar to the situation with Ki67^+^ cells, although there is a 2-fold decrease in Dcx^+^ cells at 48 h post IR, this is substantially less than observed in WT mice (Fig 5D). D. Change in the percentage of GFAP^+^Ki67^+^ cells in the ventral and dorsolateral domains up to 7 days post exposure to 2 Gy in *Atm^-/-^* mice. Although there is an apparent increase in GFAP^+^Ki67^+^ cells at 6 h post IR, the marked increased observed in WT mice at 7 days is not observed. Additionally, as for the analysis of all cells expressing Ki67, no marked transient loss is observed. These results are consistent with the notion that ATM-dependent responses promote qNSC activation. However, ATM has multiple impacts which might preclude cell growth by 7 days post 2 Gy due to ATR-dependent cell cycle arrest. Student’s t-test, ns = not significant. Underlying data can be found in the S1 Data file.
(TIF)

S6 Fig Graphical summary of the DDR in the adult and neonatal SVZ. A. Similar levels of survival are shown in cultured cells assessed by clonogenic survival assays or progenitors assessed *in vivo* by resistance to apoptosis. Black circles represent survival assessed in 1BR.3, a primary fibroblast cell line, by clonogenic survival assays. Grey circles represent survival of Dcx^+^ cells assessed by non-apoptotic cells. Fibroblasts do not undergo apoptosis even after high radiation doses. B. Model depicting the replenishment of progenitor cells by activation of quiescent stem cells in the adult SVZ in contrast to the rapid recovery of NBs in the juvenile SVZ. In the adult SVZ after 2 Gy, approximately 50% of progenitor cells die by apoptosis. The remainder lose their Ki67 proliferation marker and Dcx marker suggesting rapid proliferation arrest and differentiation. NBs are not fully recovered until 14 days post IR, which appear to arise following qNSC activation. The response of the neonatal SVZ is shown in the lower panel. Following 2 Gy, there is a similar level of apoptosis but less marked arrest of proliferation and progenitor marker loss. NBs are replenished by 48 h via continued proliferation without evidence of significant qNSC activation.
(TIF)

S1 Data Numerical data of the figures. This spreadsheet provides the numerical values used to generate the graphical data of the figures.
(XLSX)

## Abbreviations

53BP1: p53-binding protein 1
ABC: ATP-binding cassette
ATM: ataxia telangiectasia mutated
BrdU: bromodeoxyuridine
ATR: ataxia-telangiectasia-related
BSC: bulge stem cells
Casp3^+^: caspase-3^+^
CMP: common myeloid progenitors
DAPI: 4′,6-diamidino-2-phenylindole
Dcx^+^: doublecortin^+^
DDR: DNA damage response
DSB: double-strand break
EdU: ethynyl deoxyuridine
FACS: fluorescence-activated cell sorting
GFAP: Glial Fibrillary Acidic Protein
HSC: haematopoietic stem cells
IR: ionising radiation
LV: lateral ventricle
MAP2: microtubule-associated protein 2
MaSC: quiescent mammary stem cell
Mash1: mammalian Achaete-scute homologue 1
MPP: multipotent progenitors
NB: neuroblast
NHEJ: nonhomologous end-joining
ns: not significant
NSC: neural stem cell
P5: postnatal day 5
PLDR: potentially lethal damage repair
qHSC: quiescent HSC
qNSC: quiescent NSC
SA-β-Gal: β-galactosidase
SVZ: subventricular zone
TAP: transit amplifying progenitor
Tuj1: neuron-specific class III β-tubulin
TUNEL: terminal deoxynucleotidyl transferase-mediated dUTP nick end-labelling
VZ: ventricular zone
WT: wild-type
