# Supplementary figures and images for: A coordinated DNA damage response promotes adult quiescent neural stem cell activation
Source: PLoS Biol. 2017 May 10;15(5):e2001264. doi: 10.1371/journal.pbio.2001264 (PMC5424956; doi:10.1371/journal.pbio.2001264)

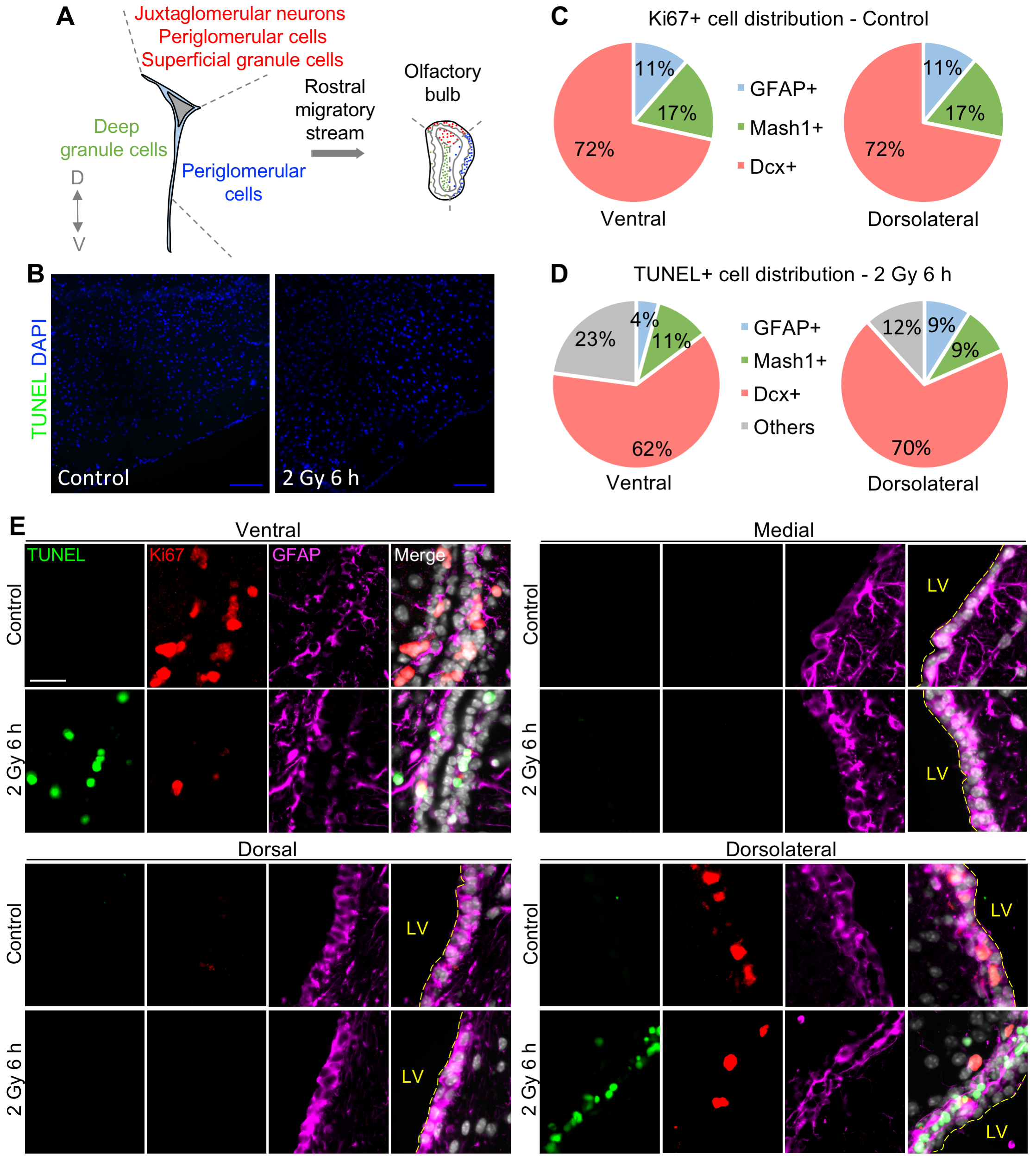

Supplement: S1 Fig — A. Schematic representation of the olfactory bulb (OB) neurons according to their spatial origin. Red, blue and green denote neurons that have originated from NSCs located in the dorsal, medial and ventral/dorsolateral sub-domains of the SVZ respectively. The medial wall predominantly generates calretin-expressing (CR+) periglomerular cells (located in the superficial layers of the OB). The ventral/dorsolateral wall produces largely deep granule cells (located in the deep layers of the OB). Whereas the dorsal wall gives rise to tyrosine hydroxylase-expressing (TH+) periglomerular and superficial granule cells in addition to a small population of juxtaglomerular glutamatergic neurons [17]. B. TUNEL staining of the isocortex in adult mice without IR or 6 h post 2 Gy. No TUNEL+ cells could be detected in the isocortex after IR demonstrating that the differentiated isocortex is resistant to apoptosis. Scale bars, 100 μm. C. Distribution of Ki67+ cells in the ventral and dorsolateral sub-domains of the lateral ventricle. D. Distribution of TUNEL+ cells in the ventral and dorsolateral sub-domains of the lateral ventricle. E. Separate color channels of the images presented in Fig 1D showing TUNEL+ cells (green), Ki67+ cells (red), GFAP+ cells (magenta), and DAPI staining (grey). Scale bars, 25 μm. Underlying data can be found in the S1 Data file. (TIF) [file pbio.2001264.s001.tif]

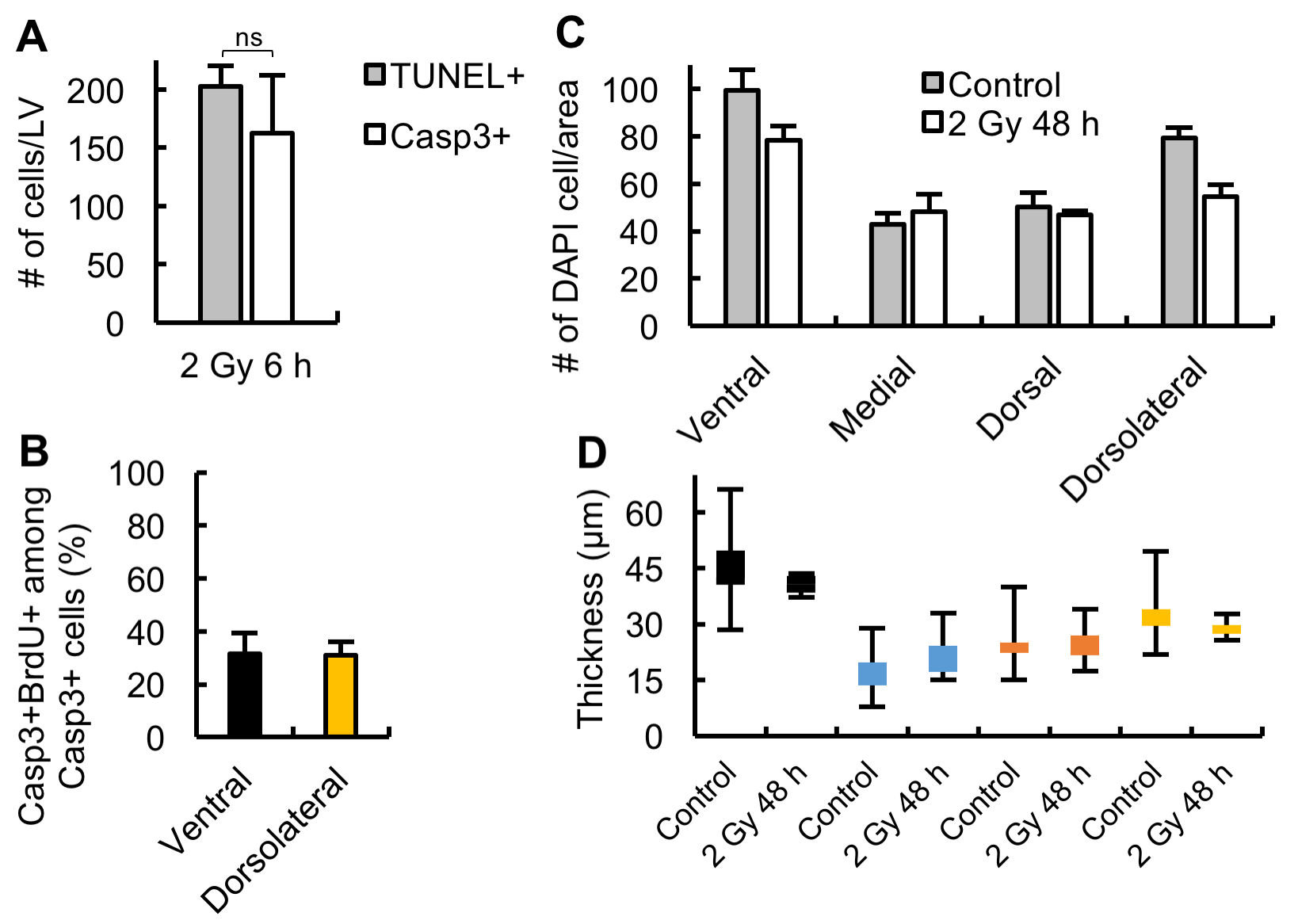

Supplement: S2 Fig — A. Quantification of the total number of TUNEL+ and Casp3+ cells per lateral ventricle (LV) at 6 h post 2 Gy. B. The percentage of Casp3+BrdU+ out of the total Casp3+ cells for the experiment depicted in Fig 2C. C. Quantification of DAPI+ cells per area in the sub-domains of the SVZ of untreated control mice and irradiated mice at 48 h following 2 Gy IR. D. Thickness of the SVZ walls in the sub-domains analysed in S2C Fig. Black, ventral; blue, medial; orange, dorsal; yellow, dorsolateral. Student’s t-test, ns = not significant. Underlying data can be found in the S1 Data file. (TIF) [file pbio.2001264.s002.tif]

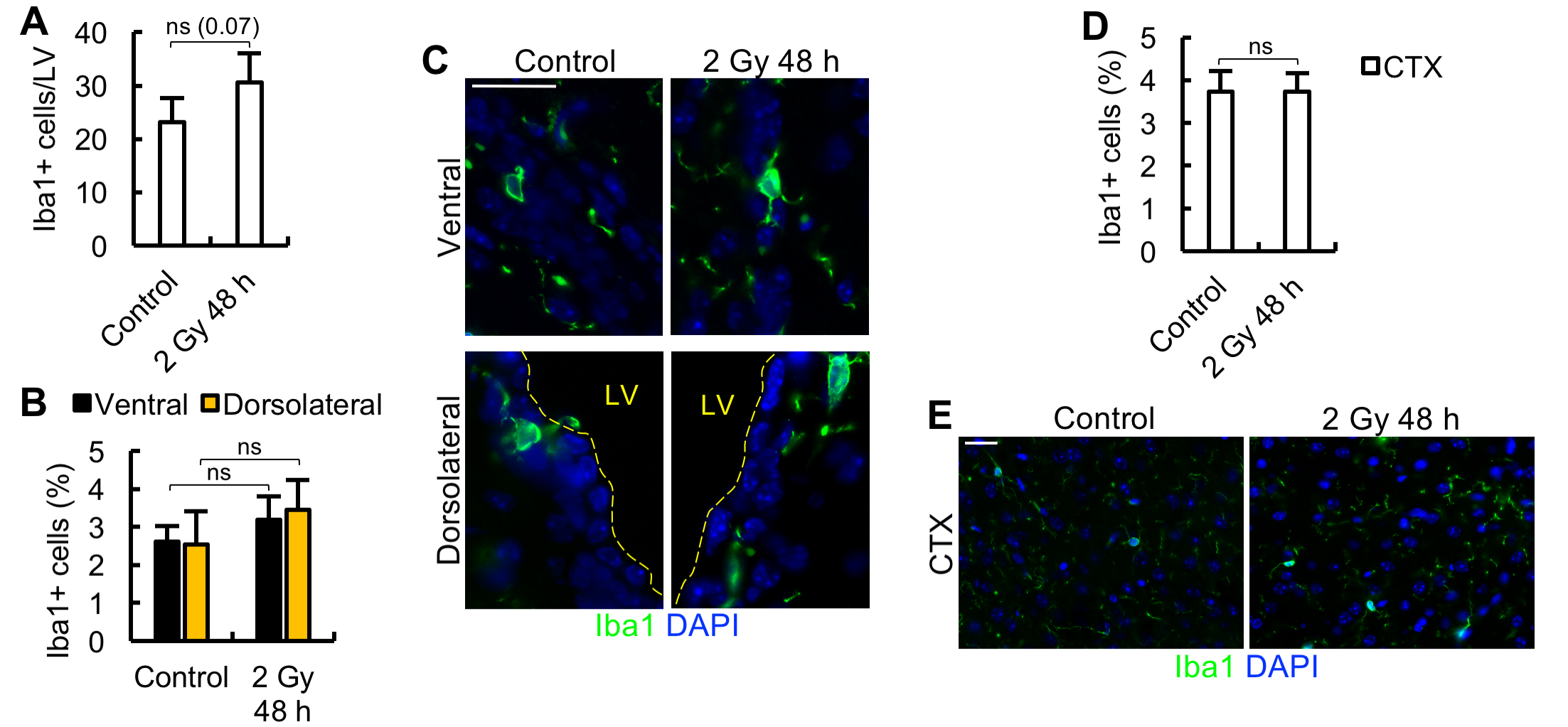

Supplement: S3 Fig — A. Quantification of the total number of Iba1+ cells per lateral ventricle (LV) of control and irradiated mice at 48 h post 2 Gy. B. Percentages of Iba1+ cells in the ventral and dorsolateral sub-domains of the SVZ as indicated above. C. Representative images of Iba1 staining from experiment shown in panel B. D. Percentages of Iba1+ cells in the differentiated isocortex (CTX) of control and irradiated mice at 48 h post 2 Gy. E. Representative images of experiments carried out in panel D. Experiments were carried out on 3 month old mice and results represent the mean ± SEM of n ≥ 3 mice for each condition. Scale bars, 25 μm. Student’s t-test, ns = not significant. Underlying data can be found in the S1 Data file. (TIF) [file pbio.2001264.s003.tif]

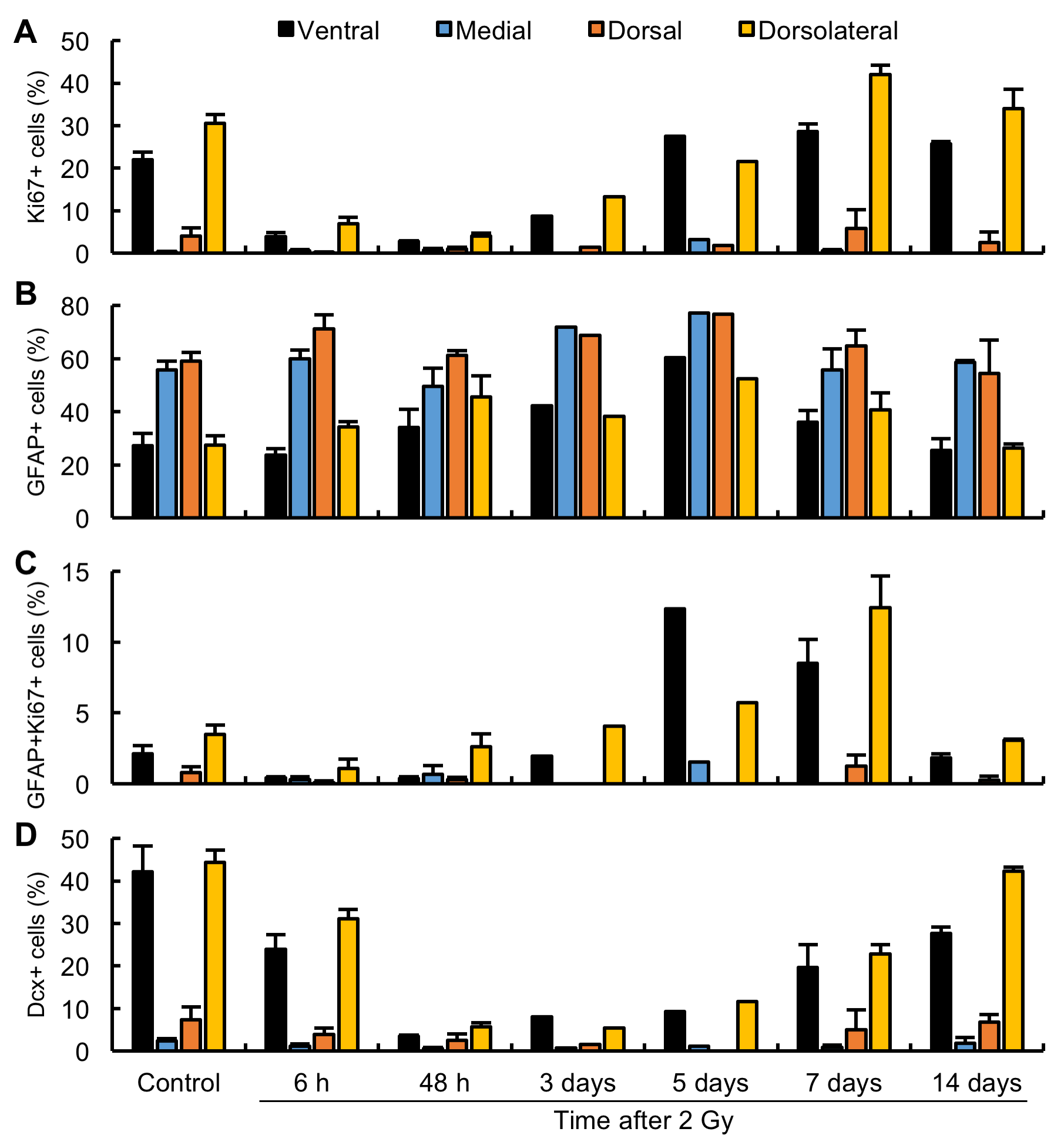

Supplement: S4 Fig — Analysis of all sub-domains for the data shown in Fig 5. In addition, data is shown for analysis of mice at 3 and 5 days post IR. Only a single mouse was quantified for each of these two time points (hence error bars are not included). The data shows quantification of (A) Ki67+ cells, (B) GFAP+ cells, (C) GFAP+Ki67+cells, and (D) Dcx+ cells. Underlying data can be found in the S1 Data file. (TIF) [file pbio.2001264.s004.tif]

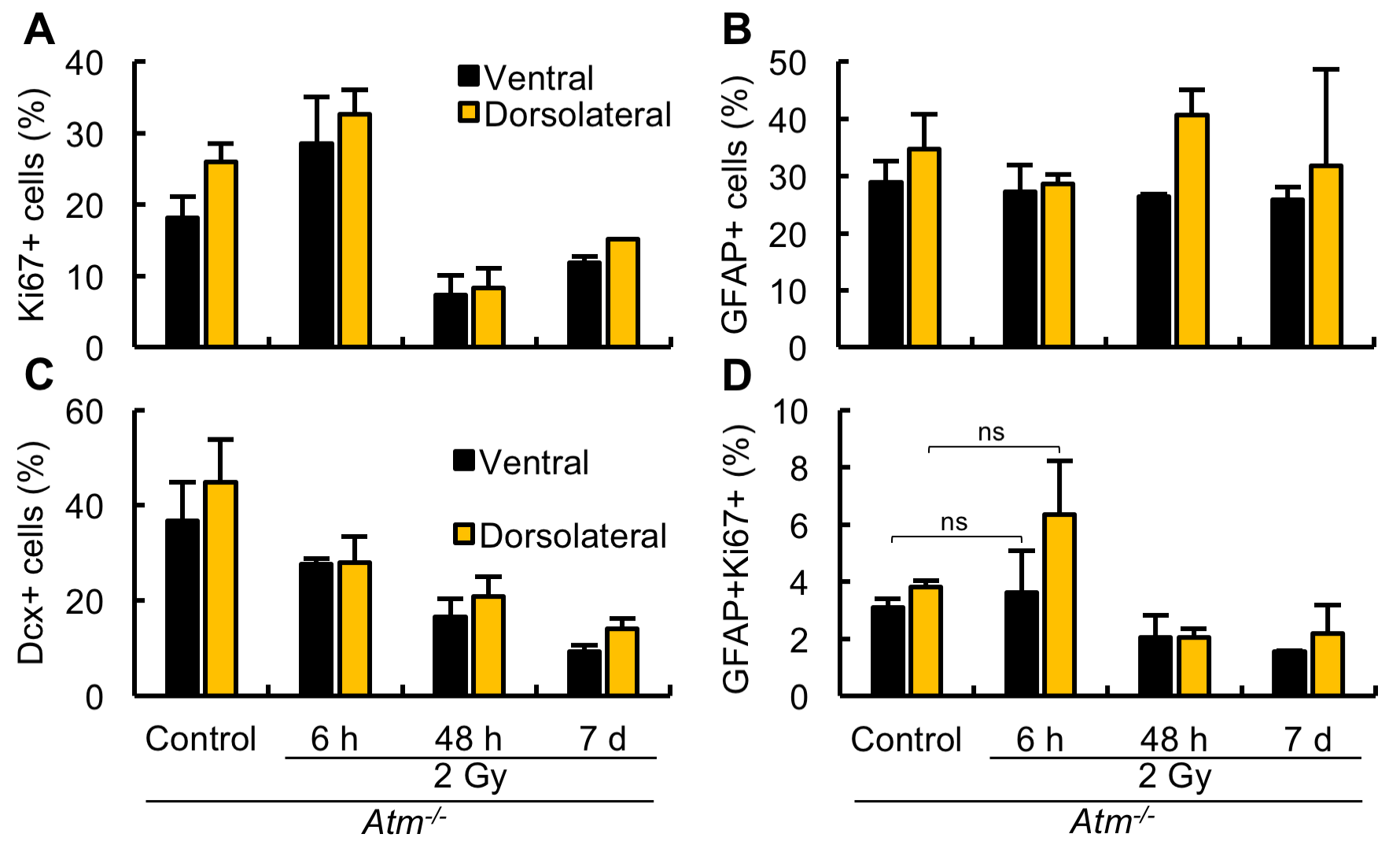

Supplement: S5 Fig — A. The percentage of Ki67+ cells in the ventral and dorsolateral domains up to 7 days post exposure to 2 Gy in Atm-/- mice. For comparison the response of WT mice is shown in Fig 5A. Notably, the number of Ki67+ cells is not reduced at 6 h post IR. By 48 h the level of Ki67+ cells is slightly reduced but remains higher than in WT mice at 48 h. Further, the number of Ki67+ cells at 7 days post IR does not increase. B. Change in the percentage of GFAP+ cells in the ventral and dorsolateral domains up to 7 days post exposure to 2 Gy in Atm-/- mice. C. Change in the percentage of Dcx+ cells in the ventral and dorsolateral domains up to 7 days post exposure to 2 Gy in Atm-/- mice. Similar to the situation with Ki67+ cells, although there is a 2-fold decrease in Dcx+ cells at 48 h post IR, this is substantially less than observed in WT mice (Fig 5D). D. Change in the percentage of GFAP+Ki67+ cells in the ventral and dorsolateral domains up to 7 days post exposure to 2 Gy in Atm-/- mice. Although there is an apparent increase in GFAP+Ki67+ cells at 6 h post IR, the marked increased observed in WT mice at 7 days is not observed. Additionally, as for the analysis of all cells expressing Ki67, no marked transient loss is observed. These results are consistent with the notion that ATM-dependent responses promote qNSC activation. However, ATM has multiple impacts which might preclude cell growth by 7 days post 2 Gy due to ATR-dependent cell cycle arrest. Student’s t-test, ns = not significant. Underlying data can be found in the S1 Data file. (TIF) [file pbio.2001264.s005.tif]

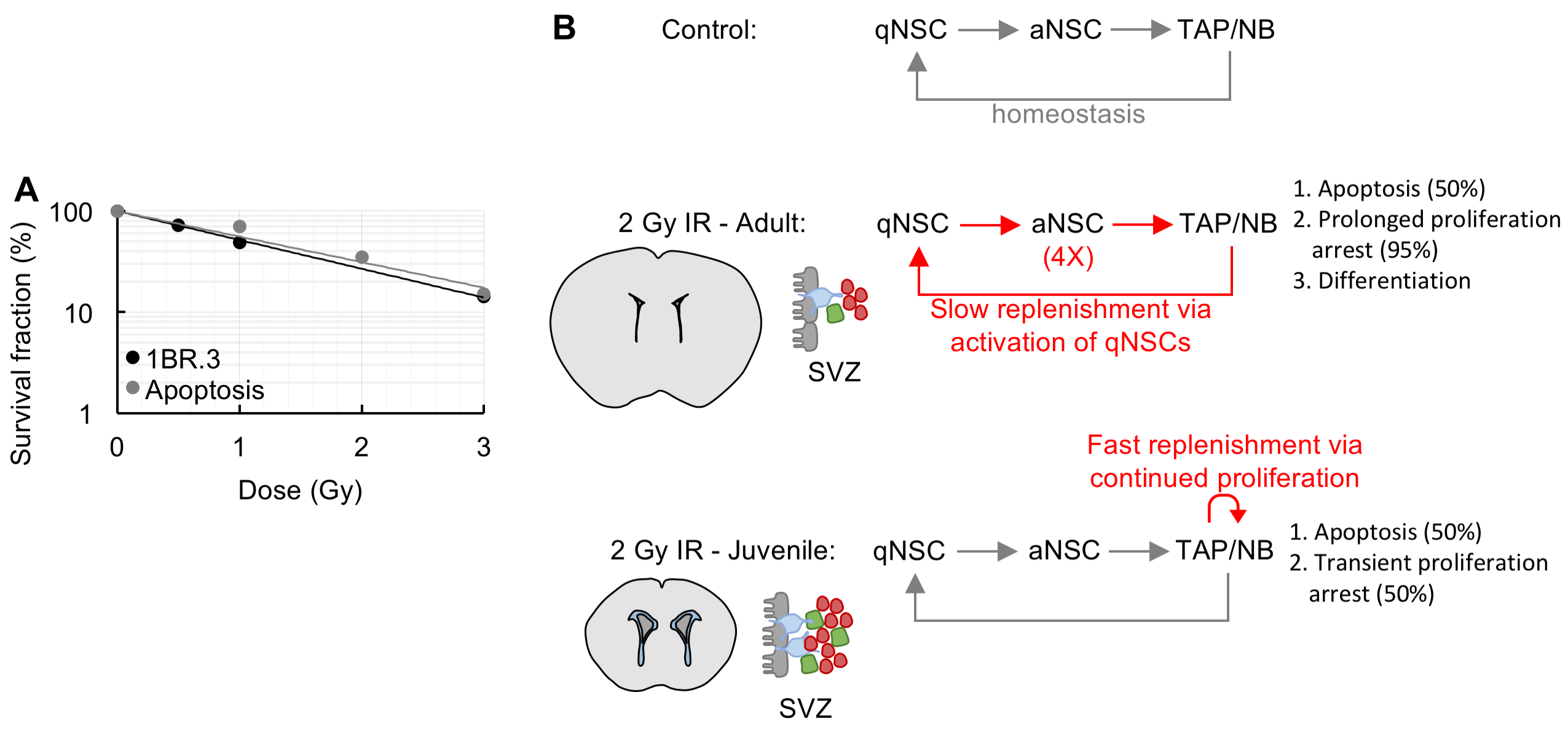

Supplement: S6 Fig — A. Similar levels of survival are shown in cultured cells assessed by clonogenic survival assays or progenitors assessed in vivo by resistance to apoptosis. Black circles represent survival assessed in 1BR.3, a primary fibroblast cell line, by clonogenic survival assays. Grey circles represent survival of Dcx+ cells assessed by non-apoptotic cells. Fibroblasts do not undergo apoptosis even after high radiation doses. B. Model depicting the replenishment of progenitor cells by activation of quiescent stem cells in the adult SVZ in contrast to the rapid recovery of NBs in the juvenile SVZ. In the adult SVZ after 2 Gy, approximately 50% of progenitor cells die by apoptosis. The remainder lose their Ki67 proliferation marker and Dcx marker suggesting rapid proliferation arrest and differentiation. NBs are not fully recovered until 14 days post IR, which appear to arise following qNSC activation. The response of the neonatal SVZ is shown in the lower panel. Following 2 Gy, there is a similar level of apoptosis but less marked arrest of proliferation and progenitor marker loss. NBs are replenished by 48 h via continued proliferation without evidence of significant qNSC activation. (TIF) [file pbio.2001264.s006.tif]
